# Mechanistic insight into complement C3 regulation during chronic HBV infection: effect on viral persistence and host immune response

**DOI:** 10.1186/s12929-026-01259-6

**Published:** 2026-05-25

**Authors:** Ayana Baidya, Debangana Dey, Shreya Mallik, Sudeshna Halder, Najma Khatun, Rambha Jha, Sarthak Nandi, Bidhan Chandra Chakraborty, Amrita Dutta, Soma Banerjee, Abhijit Chowdhury, S. K. Mahiuddin Ahammed, Simanti Datta

**Affiliations:** 1 Centre for Liver Research, School of Digestive and Liver Diseases, Institute of Post Graduate Medical Education and Research (I.P.G.M.E. & R.), 244, A.J. C. Bose Road, Kolkata, 700020 India; 2 Multidisciplinary Research Unit, Institute of Post Graduate Medical Education and Research, Kolkata, India; 3 Department of Hepatology, School of Digestive and Liver Diseases, Institute of Post Graduate Medical Education and Research, Kolkata, India

**Keywords:** Complement C3, Chronic hepatitis B virus (HBV) infection, Methylation, Histone deacetylation, Phosphorylation, Autophagy, HBV release, Monocyte defect, HBV-specific T-cell defect, Tenofovir

## Abstract

**Background:**

The complement system (CS) is central to antiviral defence, yet many viruses subvert it to escape clearance. Complement-component C3 is the key effector molecule of CS that plays diverse roles in pathogen elimination, inflammation and immune responses, but its regulation during chronic HBV infection (CHI) remains poorly understood. This study examined the mechanisms governing C3 expression in hepatocytes and immune cells during CHI and evaluated the impact of altered C3 levels on viral persistence and host immunity.

**Methods:**

C3 expression was evaluated in blood and liver-tissue samples from patients with CHI and healthy controls using ELISA and RT-PCR. HBV-transfected hepatoma cells were used to examine epigenetic regulation of C3, including promoter methylation, histone deacetylation, and transcription factor phosphorylation. Autophagy-related functions of C3 were assessed by immunofluorescence. Flow cytometry was used to determine the intracellular C3 levels in immune cells of HBV-infected patients and the influence of viral and host factors on C3 expression. Additionally, cytokine production by immune cells and the modulating effect of recombinant C3a on these cytokines was studied. The effect of Tenofovir therapy on C3 concentration was also evaluated.

**Results:**

C3 level was found to be significantly diminished in sera and liver-tissues of HBV-infected patients and HBV-expressing hepatoma cells relative to controls. HBX suppressed C3 transcription by restricting the availability of the crucial transcription factor, C/EBPβ through its promoter hypermethylation and by enhancing histone deacetylation at C3-promoter including C/EBPβ binding-sites whereas, HBV surface proteins (HBS) further blocked C/EBPβ phosphorylation essential for transcription. Functionally, C3 deficiency disrupted autophagosome-lysosome fusion and prevented autophagic degradation of viral proteins, leading to enhanced HBV release. Reduced intracellular C3 was noted in monocytes and virus-specific T cells of HBV-infected patients that could be attributed to exposure to HBS and cytokines (IL-4/IL-1β). The decline in C3 skewed the immune responses toward an anti-inflammatory phenotype with diminished pro-inflammatory activities, which were reversed by recombinant C3a. One-year of Tenofovir therapy partially restored serum but not immune-cell C3 levels.

**Conclusion:**

HBV downregulates C3 through epigenetic and signalling mechanisms, facilitating viral persistence and dampening antiviral immunity. Targeting complement regulation may represent a novel therapeutic strategy in CHI.

**Graphical abstract:**

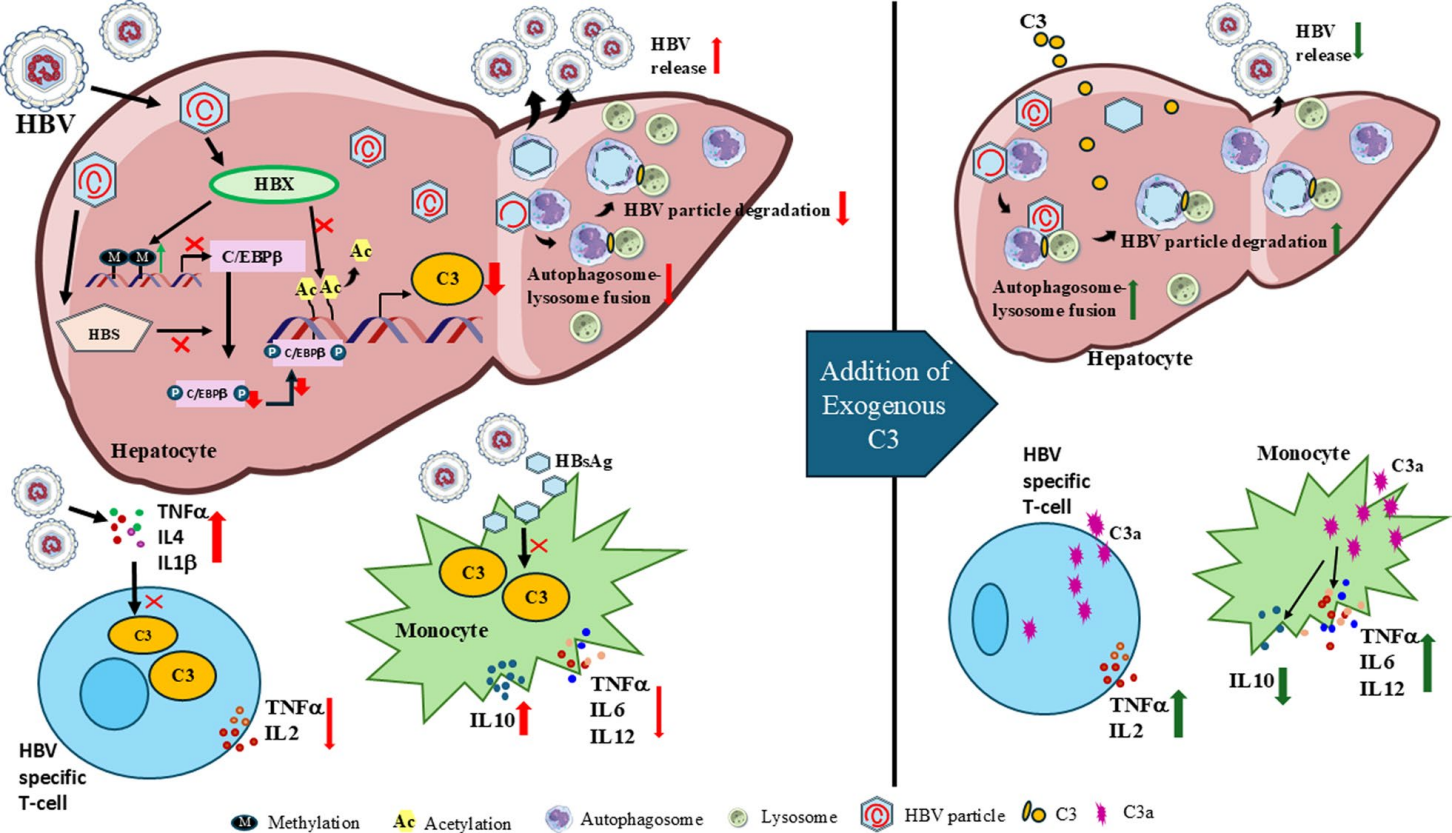

**Supplementary Information:**

The online version contains supplementary material available at 10.1186/s12929-026-01259-6.

## Background

The complement system (CS) is a crucial component of innate immunity that plays a key role in the detection and removal of invading pathogens, including viruses [1]. It can exert profound anti-viral effect via multiple mechanisms such as, opsonization and lytic destruction of virus and virus-infected cells, induction of immunoinflammatory state, regulation of chemotaxis and enhancement of virus-specific immune responses [2, 3]. While the main source of serum-circulating complement components is the hepatocytes of the liver, some complement proteins are also expressed by various immune and non-immune cell populations and these locally or intracellularly acting complement components (complosome) are critical in maintaining cellular homeostasis and effector responses [4, 5]. Among the nine major components of CS [C1-C9], complement C3, a 185 kDa glycoprotein, is most abundant both in circulation and intracellularly and is central to the complement activation cascades. All three complement activation pathways, the classical, lectin and alternative pathways, converge at the step of C3 cleavage, producing a small C3a fragment (~ 9 kDa) and a larger fragment C3b (~ 177 kDa).

While C3a promotes recruitment of immune cells and modulates inflammatory responses, C3b functions as a major opsonin and participates in the formation of C5 convertase, thereby initiating the terminal lytic phase of CS.

Beyond its extracellular functions, intracellular C3 plays important roles in autophagy, endoplasmic reticulum function, and lipid homeostasis in the hepatocytes [6]. The extrahepatic production of C3 has been observed in different immune-cells including T cells, and monocyte/macrophages, where this complement-component acts as regulators of immune response [4]. In T cells, intracellular C3 is processed by cathepsin L into C3a and C3b, which subsequently bind to their respective receptors, C3aR and CD46 to drive cytokine production [7]. Moreover, in macrophages, intracellular C3a has also been found to modulate the release of pro-inflammatory cytokines such as IL-1β [8]. CS has the ability to recognize large panoply of viruses and consequently many viruses have evolved mechanisms to evade the complement attack. For example, rhinoviruses and polioviruses, express cytosolic proteases that degrade C3 [9], Nipah and Chikungunya viruses mimic factor I-like activity that mediates C3b inactivation [10, 11], while Hepatitis C virus (HCV) had been shown to inhibit C3 convertase formation, which is crucial for C3 activation [12]. Hence a comprehensive understanding of the interplay between the virus and CS may yield deeper insights into the disease biology and help in the development of novel therapeutic approaches for management of viral infections.

Hepatitis B virus (HBV) is a small, enveloped DNA virus that selectively infects human hepatocytes and its chronic infection can progress to advanced liver diseases, including cirrhosis and hepatocellular carcinoma (HCC). The ~ 3.2 kb HBV genome comprises four overlapping open reading frames (ORFs): (i) ORF-P coding for HBV polymerase (P); (ii) pre-S1/pre-S2/S ORF encoding three envelope proteins, large/middle/small, collectively known as hepatitis B surface (HBS) proteins, with the small surface protein (HBsAg) serving as a key serological marker for infection, (iii) pre-C/C-ORF coding for core protein (HBc) along with a secretory protein, hepatitis B e antigen (HBeAg) and (iv) X-ORF encoding HBX protein [13]. The progression and pathogenesis of chronic HBV infection (CHI) are largely shaped by immune-mediated interactions between the host and the virus. The inability of the host to generate a strong and effective immune response against the virus is associated with the failure to achieve long-term viral control in CHI. Previous studies had depicted a significant reduction in the serum levels of complement C3 and C4 in chronically HBV infected patients [14]. We have recently demonstrated that HBV suppresses C9 synthesis and inhibits MAC formation [15]. However, how HBV alters C3 expression and its relevance in HBV disease pathogenesis remains largely unknown. The present study examined the effect of CHI on hepatocyte- and immune-cell derived C3 expression, deciphered the molecular mechanisms through which HBV influences C3 production and assess the consequences of altered C3 levels on HBV life cycle and virus-specific T cell and monocyte functions. This understanding will create new opportunities to develop targeted therapeutic approaches aimed at improving complement function, strengthening antiviral immune responses and preventing disease progression in patients with CHI.

## Methods

### Study subjects and samples

Treatment-naïve patients with chronic HBV infection, who had been HBsAg-positive for more than six months, were recruited for the study from the Hepatology Clinic at the School of Digestive and Liver Diseases, Institute of Post Graduate Medical Education and Research (IPGME&R), Kolkata, India. The study subjects were categorized as follows:

(i) *Immune tolerant (IT):* These patients were HBeAg-positive, had high HBV-DNA levels (> 10⁷ IU/ml), maintained normal serum alanine transaminase (ALT) levels (≤ 40 IU/L) across three consecutive follow-ups within one year prior to enrolment and exhibited minimal or no hepatic necroinflammation;
(ii) * Chronic hepatitis B (CHB):* These patients, who may be either HBeAg-positive or negative, had HBV-DNA levels > 10^4^ IU/ml, ALT levels > 40 IU/L and evidence of ongoing hepatic necroinflammation and
(iii) * Inactive carrier (IC):* These patients were HBeAg-negative, had HBV DNA levels < 2000 IU/ml and maintained ALT levels ≤ 40 IU/L on three separate measurements taken three months apart, with no biochemical, clinical, or histological signs of liver disease.

Patients with co-infection (HIV, HCV, or HDV), significant comorbidities such as diabetes mellitus, chronic alcoholism, or intravenous drug use, as well as those with evidence of malignancy, active infection, or autoimmune disorders, were excluded from the study. Additionally, HBV-uninfected healthy controls (HCs) with no history of bacterial or viral infection or acute/chronic illness in the preceding six months, along with individuals who had resolved acute HBV infection (HBsAg-negative and anti-HBc-positive), were included as the control group.

Informed written consent was obtained from all participants, or from the parents or legal guardians in the case of minors, prior to enrolment in the study. Blood samples were collected from study subjects in clot activator collection tubes for serum isolation and in EDTA pre-coated collection vials for performing immunological experiments. Liver tissue samples were obtained from selected CHB patients during ultrasound-guided routine biopsies performed as part of their diagnostic evaluation. Additionally, liver tissues from HBV-uninfected patients undergoing cholecystectomy and exhibiting normal histology were used as controls. The tissue samples were preserved in RNAlater (Invitrogen), kept at 4°C overnight to allow thorough penetration and stabilization of RNA and subsequently stored at −80°C until RNA isolation. All experimental procedures were approved by I.P.G.M.E.&R. Ethical Review Committee.

### Measurement of C3 level in patient sera

Serum C3 levels in chronically HBV-infected patients and HC were quantified using commercial ELISA kits (Elabscience) according to the manufacturer’s instructions.

### Expression of C3 in liver tissues

For RNA isolation, the frozen tissues were homogenized in TRIzol reagents (Thermo Fisher Scientific) followed by a second round of TRIzol treatment and then the RNA was isolated. cDNA was prepared from the RNA by reverse transcription using RevertAid Reverse Transcriptase enzyme and Random Hexamer primer (Thermo Fisher Scientific). The expression levels of C3 mRNA was evaluated by quantitative real-time PCR using SYBR Green Master Mix (Applied Biosystems) and C3 sequence-specific primers [Supplementary (S) Table S1].

### Cell culture

Human hepatoma Huh7 cells and the HBV-transfected stable cell line HepG2.2.15 were maintained in Dulbecco’s modified Eagle’s medium (DMEM) supplemented with 10% FBS. The HBV-susceptible HepG2^hNTCP^ line, expressing the human sodium taurocholate co-transporting polypeptide (NTCP), was cultured in DMEM supplemented with 10% FBS and 1% L-glutamine. The Huh7 cell line was authenticated through Short Tandem Repeat (STR) profiling at the National Centre for Cell Science (NCCS), Pune, India. The HepG2.2.15 and HepG2^hNTCP^ cell lines were a generous gift from Prof. Shyam Kottilil of the Institute of Human Virology, University of Maryland.

### Plasmids

The plasmids pJET1.2-HBV, containing full-length HBV genome of genotype D, pEGFPN1-HBs, encoding HBV-surface and pcDNA3.1/myc-His(B)-HBV-P, encoding HBV-polymerase were available in the laboratory [15]. The plasmids pCMV-HBc and pcDNA3.1/myc-His(B)-HBx encoding HBV-core and HBX proteins respectively were gifted by Prof. Soma Banerjee, I.P.G.M.E.&R, Kolkata [16] while ptfLC3 having mRFP and EGFP expressing genes fused with LC3 was gifted by Dr. Dipanjan Ghosh, National Institute of Pharmaceutical Education and Research, Kolkata.

### Transfection

Sixteen hours prior to transfection, Huh7 cells were seeded in 6-, 12- or 24-well plates at densities of 3 × 10^5^, 2 × 10^5^ and 1 × 10^5^ cells per well, respectively, depending on experimental requirements. The full-length linear HBV-monomer was excised from pJET1.2-HBV by Sap*I* digestion at 37 °C for 12 h, followed by gel purification. The purified HBV-monomer or plasmids containing specific HBV ORFs were individually transfected into Huh7 cells using Lipofectamine 3000 (Invitrogen). Untransfected or empty vector-transfected Huh7 cells served as controls. Six hours post-transfection, the medium was replaced with fresh DMEM supplemented with 10% FBS and cells were cultured for 72 h at 37°C in a humidified incubator with 5% CO₂. Cells were then harvested and culture supernatants were collected and stored for subsequent analyses. All experiments were conducted in triplicate and independently replicated at least three times.

### HBV particle production and infection

HepG2.2.15 cells were cultured for 4 days, after which HBV-containing culture supernatant was collected and viral titer, expressed as genome equivalents per milliliter, was quantified by real-time PCR [15]. Then HepG2^hNTCP^ cells were infected with HBV at 100 genome equivalents per cell in the presence of 8% polyethylene glycol (PEG 8000) and 2.5% dimethyl sulfoxide (DMSO) and after seventy-two hours, the cells were harvested for subsequent analyses.

### Real-time PCR

Total RNA was extracted from transfected/infected and control Huh7 and HepG2^hNTCP^ cells using TRIzol reagent (Invitrogen) and cDNA was synthesized by reverse transcription as described previously. Quantitative real-time PCR was performed using SYBR Green Master Mix (Applied Biosystems) to determine the mRNA expression levels of complement component C3, other relevant host genes and HBV pregenomic RNA (pgRNA), utilizing gene-specific primer pairs (Table S1). All samples were analyzed in technical triplicate across at least three independent biological replicates, with expression levels normalized to 18S rRNA.

### Immunofluorescence

HBx- and HBs-transfected Huh7 cells were cultured as monolayers on poly-L-lysine-coated coverslips in 6-well plates for 72 h in presence or absence of 10 ng/ml recombinant C3 protein (Prospec). Following fixation with 4% paraformaldehyde, cells were washed with ice-cold TBS and antigen retrieval was performed using a 0.05% trypsin-calcium chloride solution (0.5% trypsin, 1% calcium chloride, pH 7.8) at 37°C. For immunostaining, cells were permeabilized with 0.025% Triton X-100 in TBS and blocked for 2 h with 1% BSA. The cells were then incubated with an anti-human C3-AF647 primary antibody (1:500; Santa Cruz Biotechnology), mouse anti-human anti-HBX (1:500; Invitrogen) or mouse anti-human anti-HBsAg antibody (1:500; MyBiosource) for 1 h at 37°C. Nuclei were counterstained and mounted using ProLong™ Gold Antifade with 4’,6-diamidino-2-phenylindole (DAPI) (Cell Signaling Technology). Fluorescent images were acquired using a Leica THUNDER Imager at 40X magnification. For C3 localization, z-stack imaging was performed at 40X magnification, and the acquired image stacks were analyzed using sum projection in ImageJ.

### Cloning of promoter region

The pGL3-C3-Prom reporter construct was generated by amplifying the C3 promoter region (−1807 bp to +58 bp relative to the transcription start site) from the genomic DNA of Huh7 cells [17]. PCR was carried out using specific primer pairs (Table S1) designed with *Kpn*I and *Xho*I restriction sites at their respective 5′ ends. The amplified fragment was subsequently digested with these enzymes and directionally cloned into the *Kpn*I/*Xho*I sites of the pGL3-Basic vector, with the final construct verified by DNA sequencing.

### Treatment of HBx transfected Huh7 cells with 5-Aza-2’-deoxycytidine or Trichostatin A

To investigate the potential role of DNA methylation or histone deacetylation in HBX-mediated downregulation of C3 expression, HBx-transfected Huh7 cells were treated with either 5-Aza-2’-deoxycytidine (a DNA methylation inhibitor) or Trichostatin A (TSA) (a histone deacetylation inhibitor), with untreated transfected cells serving as the experimental control. 5-Aza-2’-deoxycytidine, a cytosine analogue is unstable and gets incorporated in the DNA during replication, thus it was added to the cells before transfection at the time of seeding the cells on culture plate and the culture medium was replaced every 24 h with fresh medium containing 5-Aza-2’-deoxycytidine (5 μM) for a duration of 72 h post-transfection. TSA is chemically more stable so TSA (0.1 μM) was added to the cells 6 h post-transfection and maintained for 72 h. The cells were harvested after 72 h of transfection and the total RNA was extracted and cDNA was prepared as before. The mRNA expression of both C3 and C/EBPβ were analyzed from 5-Aza-2’-deoxycytidine treated and corresponding untreated cells and the expression of C3 was measured in TSA treated/untreated cells by Real-time PCR.

### Dual luciferase reporter assay

To assess C3 promoter activity, Huh7 cells were co-transfected with the pGL3-C3-Prom construct, pcDNA3.1myc-His(B)-HBx and the pRL-CMV Renilla Luciferase Reporter vector (Promega) as an internal control. After 72-h incubation, both firefly and Renilla luciferase activities were quantified sequentially using the Dual-Luciferase Reporter Assay kit (Promega) on a GloMax luminometer.

### Western blot analysis

To determine the expression of total and phospho-C/EBPβ, Western blotting was performed using protein extracts from transfected Huh7 cells. Total C/EBPβ (cytoplasmic and nuclear) was measured from total cell lysate, while phospho-C/EBPβ was assessed in the nuclear fraction.

To prepare total cell lysates, HBx/HBs-transfected Huh7 cells were harvested and lysed in RIPA buffer (150 mM NaCl, 1% NP40, 0.5% sodium deoxycholate, 0.1% SDS, 50 mM Tris, pH 8.0) supplemented with a 1X protease inhibitor cocktail (Roche). The lysates were centrifuged and supernatants stored at  −80°C. For nuclear protein extraction, HBs-transfected cells were washed with ice-cold calcium-free PBS and resuspended in 1X hypotonic lysis buffer [10 mM Tris (pH 7.4), 10 mM NaCl, 3 mM MgCl_2_] containing 1X protease inhibitor, 1X phosphatase inhibitor (Sigma), 0.5 mM DTT, and 0.05% NP-40. After centrifugation, the cytoplasmic fraction was removed. Nuclear pellets were washed and resuspended in nuclear extraction buffer (20 mM HEPES, pH 7.4, 420 mM NaCl, 1.5 mM MgCl_2_, 0.2 mM EDTA, 25% v/v glycerol, DTT, protease inhibitor). The samples were incubated on ice for 30 min with intermittent mixing followed by centrifugation. The nuclear extract was collected and stored at −80°C.

Protein concentrations were quantified using the Bradford assay (Sigma-Aldrich) and the proteins were then resolved by SDS-PAGE, electroblotted onto PVDF membranes and blocked with 5% BSA for 1 h. Subsequently the membranes were incubated overnight at 4°C with the following primary antibodies (1:500) as required: mouse anti-C/EBPβ (Santa Cruz and Abclonal), rabbit anti-p-C/EBPβ (Cell Signaling), mouse anti-α-tubulin (Thermo Scientific), or mouse anti-H3 (Santa Cruz). The membranes were further incubated with HRP-conjugated anti-mouse IgG or anti-rabbit IgG (1:1000) for 1 h at room temperature. Blots were developed using SuperSignal West Pico substrate (BioRad) and imaged with ChemiDoc (BioRad). Band intensities were analysed by densitometry using ImageJ. α-Tubulin and histone H3 served as loading controls for total and nuclear proteins, respectively.

### Chromatin immunoprecipitation (ChIP) assay

ChIP was conducted in Huh7 cells transfected with either pcDNAmyc-His(B)-HBx or an empty vector control, utilizing the EpiQuik ChIP kit (EpigenTek) [15]. As per the manufacturer’s protocol, initially, assay strips were coated with specific antibodies against C/EBPβ or H3K9Ac (Santa Cruz Biotechnology), while non-immune IgG was employed as a negative control for nonspecific binding. The harvested cells were treated with 1% formaldehyde to crosslink the chromatin followed by termination of crosslinking using 1.25 M glycine. The cells were then incubated in lysis buffer supplemented with 1X protease inhibitor cocktail (Roche). The crosslinked DNA-protein complexes were then sheared by sonication into fragments and added to the strips coated with respective antibodies for immunoprecipitation. From the sonicated DNA–protein complexes, 5% input was removed as reference. After incubation with the antibodies, the cross linking was reversed and the precipitated DNA was purified. The DNA obtained from each test sample was then quantified by Real-time PCR using primer pairs specific for C3 promoter (Table S1). The result was shown as percent of DNA recovered relative to the input and normalization of the data was performed using the negative control values.

### Knockdown of C/EBPβ

An antisense oligonucleotide targeting exon 1 of C/EBPβ (C/EBPβ ASO) (Supplememtary information Table S1) was designed based on the C/EBPβ gene sequence retrieved from the UCSC Genome Browser. Huh7 cells were co-transfected with pGL3-C3-Prom plasmid and C/EBPβ ASO or scrambled oligo to assess relative C3 promoter activity by Luciferase assay. In parallel, C3 mRNA expression was quantified by real-time PCR in Huh7 cells transfected with C/EBPβ ASO or scrambled oligo.

### Analysis of CpG island distribution

Putative CpG islands in the promoter regions of C3 and C/EBPβ were analyzed using online tool Methprimer [18] where the promoter sequences were entered and the prediction of presence or absence of CpG islands in the provided sequence was obtained. The software identified CpG island by using a threshold value of more than 50% CG content.

### Bioinformatic analysis of transcription factors binding at C3 promoter

Transcription factor (TF) binding sites within the C3 promoter region were predicted bioinformatically using the PROMO and TFBIND software, applying a stringency threshold of < 5% dissimilarity. Candidate TFs were further validated for physiological relevance using the Human Protein Atlas to confirm their expression levels in human liver tissue. Based on these criteria, selected TFs were prioritized for subsequent in vitro functional assays to evaluate their interaction with the C3 promoter.

### Bisulfite Sequencing

To assess the methylation status of the C/EBPβ promoter, genomic DNA was extracted from Huh7 cells transfected with pcDNA-HBx or empty vector using a DNA extraction kit (FAVORGEN Biotech Corp). The isolated DNA was subjected to bisulfite conversion and purification using the EZ DNA Methylation kit (ZYMO RESEARCH) [15]. A segment of the C/EBPβ promoter (nt − 205 to + 74) was amplified from the bisulfite-modified DNA using a bisulfite-specific primer pair (Table S1) and cloned into the pJET1.2 blunt vector. For each transfection group, eight independent clones were selected and analyzed by DNA sequencing to determine the precise methylation profile.

### Measurement of HBV DNA load in cell culture supernatant

HBV transfected Huh7 cells were treated with different doses of recombinant C3 protein (5 ng/ml and 10 ng/ml) and after 72 h, the cell culture media was collected. HBV DNA was isolated from the cell culture media using QIAamp DNA Blood mini kit and then quantified by Real time PCR amplification using SYBR green master mix (Thermofisher) and HBV-specific primer pairs F5/R4 (Table S1).

### Autophagosome-lysosome fusion

To investigate the impact of HBX-mediated C3 downregulation on autophagosome-lysosome fusion, Huh7 cells were co-transfected with full-length HBV or pcDNA3.1/myc-His(B)-HBx and mRFP-GFP-LC3 (ptfLC3) plasmids and the cells were cultured on poly-L-lysine-coated coverslips in 6-well plates, either in the presence or absence of C3 (10 ng/ml). After 48 h, cells were washed with TBST (1 × TBS + 0.025% Triton X-100) and the coverslips were mounted on slides using ProLong Gold Antifade reagent with DAPI. Autophagosome-lysosome puncta were imaged using a Thunder Imager (Leica) at 40 × magnification.

### Accumulation of LC3 and HBS in transfected Huh7 cells

HBx- or full-length HBV- transfected Huh7 cells (3 × 10^5^ cells/well) were grown on poly-L-lysine coated coverslips in 6-well plate in presence or absence of 10 ng/ml recombinant C3 protein (Prospec). Immunofluorescence assay was performed by staining the cells with 1:500 dilution of rabbit anti-LC3B antibody (Abcam) (primary antibody against autophagic protein LC3B) or 1:1000 dilution of mouse anti-HBsAg antibody (My Biosource), followed by incubation with 1:1000 dilution of goat anti-rabbit IgG-AF488 (Thermo Fisher Scientific) and donkey anti-mouse IgG-AF647 (Life Technologies) secondary antibodies respectively. Finally, the accumulation of LC3B or HBS in the transfected Huh7 cells were visualized by Thunder imager (Leica).

### Determination of intracellular C3 in monocytes and HBV-specific CD4^+^/CD8^+^ T cells

To evaluate intracellular C3 expression in HLA-DR⁺CD14⁺ monocyte population, freshly collected EDTA-anticoagulated blood from different groups of treatment-naïve chronically HBV infected patients and HC was first stimulated with lipopolysaccharide (LPS) for 1 h, followed by treatment with Brefeldin A for 3 h to inhibit protein secretion. Cells were then surface stained with anti-HLA-DR-V500 and anti-CD14-FITC and subjected to erythrocyte lysis using FACS Lysing Solution (BD Pharmingen). Subsequently, the cells were fixed and permeabilized using the Cytofix/Cytoperm kit (BD Biosciences) and stained with Anti-C3-AF647. Samples were washed and analyzed on a FACSVerse flow cytometer (BD Biosciences), with fluorescence minus one (FMO) controls included in each set to reduce background signal. Data analysis was performed using FCS Express De Novo software.

To assess C3 production by HBV-specific T-cells, PBMCs were isolated from chronically HBV-infected patients across different disease phases and from acute HBV patients who had successfully resolved the infection, serving as controls. Briefly, 2 × 10⁶ PBMCs were stimulated with a set of 15-mer overlapping peptides (OLP) spanning HBV genotype D core protein (5 μg/ml; Mimotopes) for 5 days at 37 °C. On day 4, cells were restimulated with OLPs (5 μg/ml) and treated with Brefeldin A (1 μg/ml) overnight to block protein secretion. On day 5, cells were harvested and surface-stained with anti-CD3-PE-Cy7, anti-CD4-FITC and anti-CD8-PerCP. Following washing, fixation and permeabilization using the BD Cytofix/Cytoperm kit, the cells were stained intracellularly with anti-IFN-γ-BV421 and anti-C3-AF647 antibodies before acquisition on a BD FACSVerse flow cytometer.

### Gating strategy of C3 expressing monocytes and HBV-specific T cells

To define the monocyte population for flow cytometric analysis, cells were first identified by gating the monocyte and adjacent lymphocyte populations based on their forward (FSC) and side scattering (SSC) profiles. From this initial gate, the total monocyte fraction was selected based on the co-expression of CD14 and HLA-DR. Subsequently, the levels of intracellular C3 were quantified within this gated CD14^+^HLA-DR^+^ population (Supplementary Fig. S1). Conversely, to identify C3-expressing HBV-specific CD4⁺ and CD8⁺ T cells, lymphocytes were first gated based on their forward and side scatter properties. Within this gated population, CD3⁺ T cells were selected and further subdivided into CD3⁺CD4⁺ and CD3⁺CD8⁺ subsets. HBV-specific T cells were then identified by their capacity for intracellular IFN-*γ* production, allowing for the subsequent frequency analysis of IFN-*γ*^+^*C*3^+^ cells within both the helper and cytotoxic T cell compartments (Fig. S2).

### Serum cytokine and HBsAg quantification

Different cytokines present in the serum samples of the study subjects were measured using BD CBA Human Th1/Th2/Th17 Cytokine Kit (BD Bioscience). The concentration of serum HBsAg in the chronically HBV infected patients were quantified using Abbott Architect i1000sr platform.

### Treatment of sorted monocytes from healthy individuals with recombinant (r) HBsAg

Monocytes were isolated from PBMCs of HC using anti-CD14-coated magnetic beads and processed on an AutoMACS separator, achieving a purity of > 95%. For subsequent functional assays, the sorted CD14⁺ monocytes were cultured in RPMI medium (Gibco, MD, USA) supplemented with 10% heat-inactivated FBS for 48 h, either in the presence of rHBsAg (20 μg/ml), β-gal (20 μg/ml), or left untreated. The cells were then harvested, surface-stained with anti-HLA-DR-BV421 and anti-CD14-FITC, followed by fixation, permeabilization and intracellular staining with anti-C3-AF647 and analyzed via flow cytometry.

### Treatment of HBV-specific T cells with specific inhibitor against host cytokines

PBMC of CHB patients were stimulated with OLP of HBV core antigen (5 μg/ml) in presence or absence of anti-IL-4, anti-TNFα or anti-IL-1β antibody and cultured in RPMI supplemented with 10% autologous serum for 5 days followed by enumeration of C3 expression on CD4^+^ IFN-γ^+^ or CD8^+^ IFN-γ^+^ T-cells.

### Modulation of cytokine production of monocytes and HBV-specific CD4^+^/CD8^+^ T cells by recombinant C3a

To evaluate the immunomodulatory effect of C3a on monocyte cytokine production, PBMCs from CHB patients were isolated and cultured for 24 h with or without rC3a protein at different concentrations (5, 10 and 20 ng/ml). Following incubation, the cells were surface-stained with anti-CD14-FITC and anti-HLA-DR-BV421 monoclonal antibodies, fixed and permeabilized using the Cytofix/Cytoperm kit and stained intracellularly with anti-TNF-*α*-APC, anti-IL-6-PE, anti-IL-12-PE and anti-IL-10-APC. After washing, samples were acquired and analyzed by flow cytometry.

To investigate the role of C3 in regulating cytokine production by HBV-specific T cells, PBMCs from CHB patients were isolated using LSM 1077 density gradient centrifugation. The cells were stimulated with OLPs of the HBV core antigen, as previously described, in the presence or absence of varying doses of rC3a. Subsequently, the cells were harvested and surface-stained with anti-CD3-PE-CY7, anti-CD4-FITC and anti-CD8-PerCP. After fixation and permeabilization, intracellular staining was performed with anti-IFN-*γ*-BV421 and anti-IL-2-PE. The production of these cytokines by CD4^+^ and CD8^+^ T cells was then quantified via flow cytometry.

### Assessment of C3 levels in serum and within monocytes and HBV-specific CD4^+^/CD8^+^ T cells following Tenofovir therapy

Blood samples were collected from 10 CHB patients treated with Tenofovir (300 mg daily) at the initiation of therapy (= baseline) and after 12 months of therapy and the serum C3 level, intracellular C3 stores in monocytes and HBV specific CD4^+^ and CD8^+^ T cells along with the serum HBV-DNA, ALT levels, HBsAg load and cytokines were determined at both time points and compared.

### Statistical analysis

Data were expressed as mean ± standard deviation (SD). Comparison between groups was done by Paired Student’s t tests, one-way ANOVA followed by Tukey’s Multiple Comparison Test and Repeated Measures ANOVA to determine statistical significance. Linear regression was performed for correlation analysis. Statistical analysis was performed using GraphPad Prism Software. For all tests, *p* < 0.05 was considered statistically significant.

## Results

### Reduction in C3 level in sera and liver tissues of chronically HBV-infected patients and in HBV-transfected/infected human hepatoma cells

**Fig. 1:**
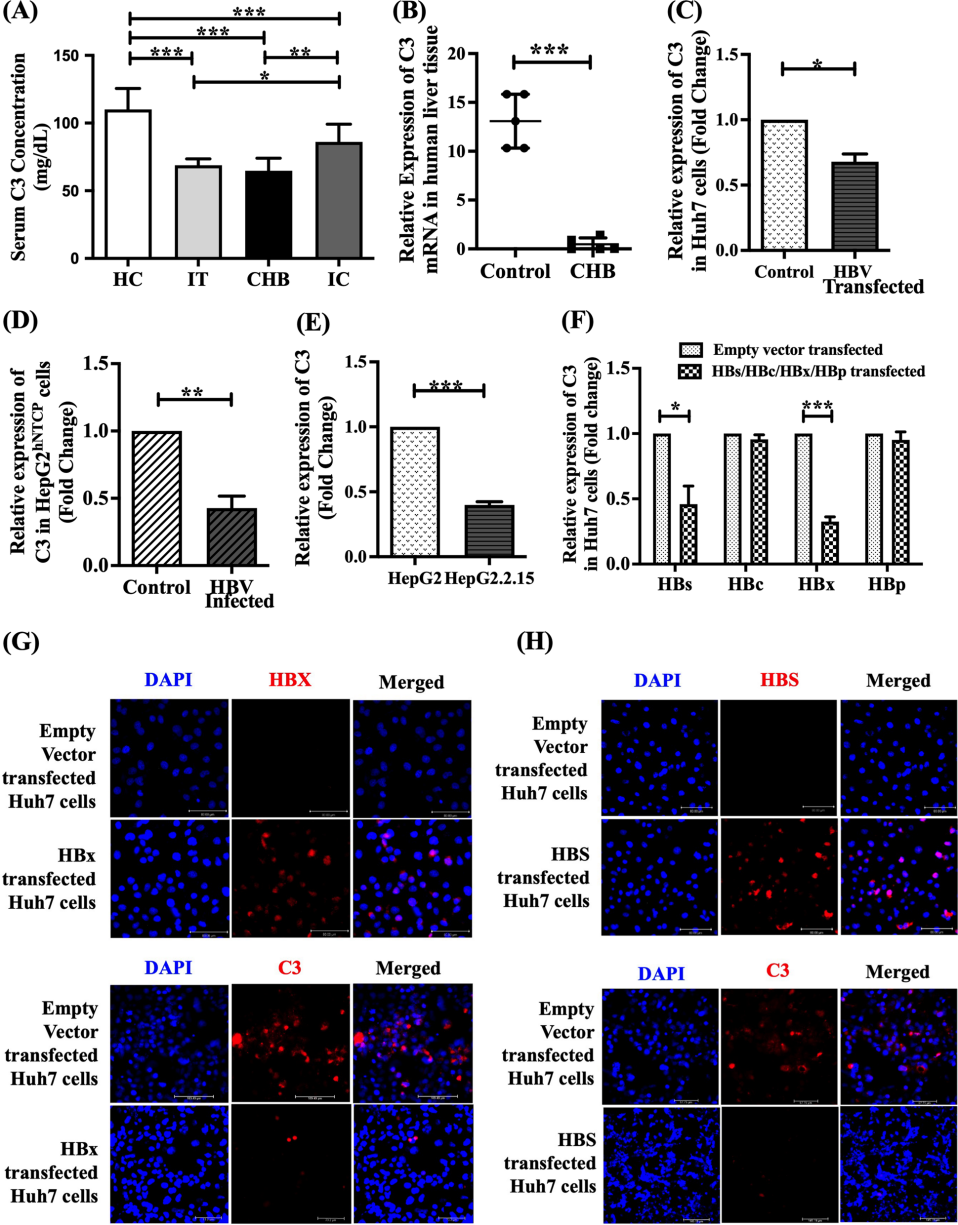
HBV reduces complement component C3. **A** Serum levels of C3 in immune tolerant (IT) (n = 10), chronic hepatitis B (CHB) (n = 27), inactive carrier (IC) (n = 22) and healthy control (HC) (n = 20) detected by ELISA. **B** Quantitative PCR analysis of C3 mRNA expression in liver biopsy tissues of CHB patients (n = 5) compared to controls (n = 5). **C**–**F** Relative C3 mRNA expression in (**C**) Huh7 cells transfected with full length HBV genome relative to untransfected cells (control), (**D**) HBV infected HepG2^hNTCP^ cells compared to uninfected HepG2^hNTCP^ cells (control), (**E**) HBV stably transfected HepG2.2.15 cells compared to normal HepG2 cells (control) and (**F**) Huh7 cells transfected separately with plasmids encoding HBV surface proteins (HBs), HBV core (HBc), HBV X (HBx) or HBV polymerase (HBp). **G**, **H** Representative immunofluorescence images at 40X magnification showing HBX (red) and C3 (red) in HBx-transfected Huh7 cells (**G**), and HBS (red) and C3 (red) in HBs-transfected Huh7 cells (**H**). In both G and H the results are compared with their respective empty vector transfected cells. The nucleus was counterstained with DAPI (blue). mRNA expression was normalized with endogenous 18S ribosomal RNA value. mean ± SD. In case of C-F, data represents mean ± SD from three independent experiments. For A, Statistical significance was assessed by one-way ANOVA with Tukey’s Multiple Comparison post-hoc Test. Paired *t*-tests were performed for comparisons of paired groups in B-F. *p < 0.05, **p < 0.005, ***p < 0.0001

To investigate the impact of HBV on complement C3 synthesis, we first measured serum C3 concentrations using ELISA in chronically HBV-infected patients representing different phases of chronic HBV infection (CHI) namely immune tolerant (IT), chronic hepatitis B **(**CHB) and inactive carrier (IC) and compared them to those of healthy controls (HC). The clinical and demographic profiles of all study subjects are provided in Table S2. Serum C3 was found to be significantly low in IT and CHB relative to IC and HC (Fig. 1A). Moreover, when compared between IC and HC, C3 level was less in IC than HC. In parallel, we observed a profound decrease in C3 mRNA expression in liver biopsy tissues of CHB patients than uninfected individuals (Fig. 1B). We further determined the expression of C3 in Huh7 cells transfected with full-length HBV by Real-time PCR. As depicted in Fig. 1C, a marked diminution in C3 expression (~ 1.5-fold) was noted in HBV-transfected Huh7 cells in comparison to untransfected cells. In addition, we also detected a significant downregulation of C3 expression (~ 2.4-fold) in HepG2^hNTCP^cells infected with cell culture-derived HBV produced by stable HBV-replicating cells, HepG2.2.15 as compared to uninfected HepG2^hNTCP^cells (Fig. 1D). The HepG2.2.15 cells also exhibited ~ 2.5-fold reduced C3 expression relative to HepG2 cells (Fig. 1E).

### HBX and HBS inhibit C3 synthesis

To identify the viral factors that could supress C3 synthesis in hepatocytes, Huh7 cells were separately transfected with plasmids expressing different HBV proteins and the expression of C3 was evaluated by Real-time PCR. C3 transcript level was found to be significantly low in HuH7 cells transfected with plasmids encoding HBX as well as HBS by about 3-folds and 2.2-folds respectively in comparison to cells transfected with corresponding empty-vectors (Fig. 1F). In contrast, Huh7 cells expressing other HBV proteins, HBV core and HBV polymerase showed no significant change in the amount of C3 mRNA with respect to control. The downregulatory effect of HBX and HBS on C3 expression was further confirmed at protein levels by immunofluorescence analysis using fluorophore-conjugated anti-C3 antibody in Huh7 cells expressing these viral proteins relative to control cells (Fig. 1G, H). The expression of HBX and HBS in the respective transfected cells were also confirmed by immunofluorescence assay (Fig. 1G, H).

### HBX induced DNA hypermethylation and histone deacetylation contribute to C3 downregulation

**Fig. 2:**
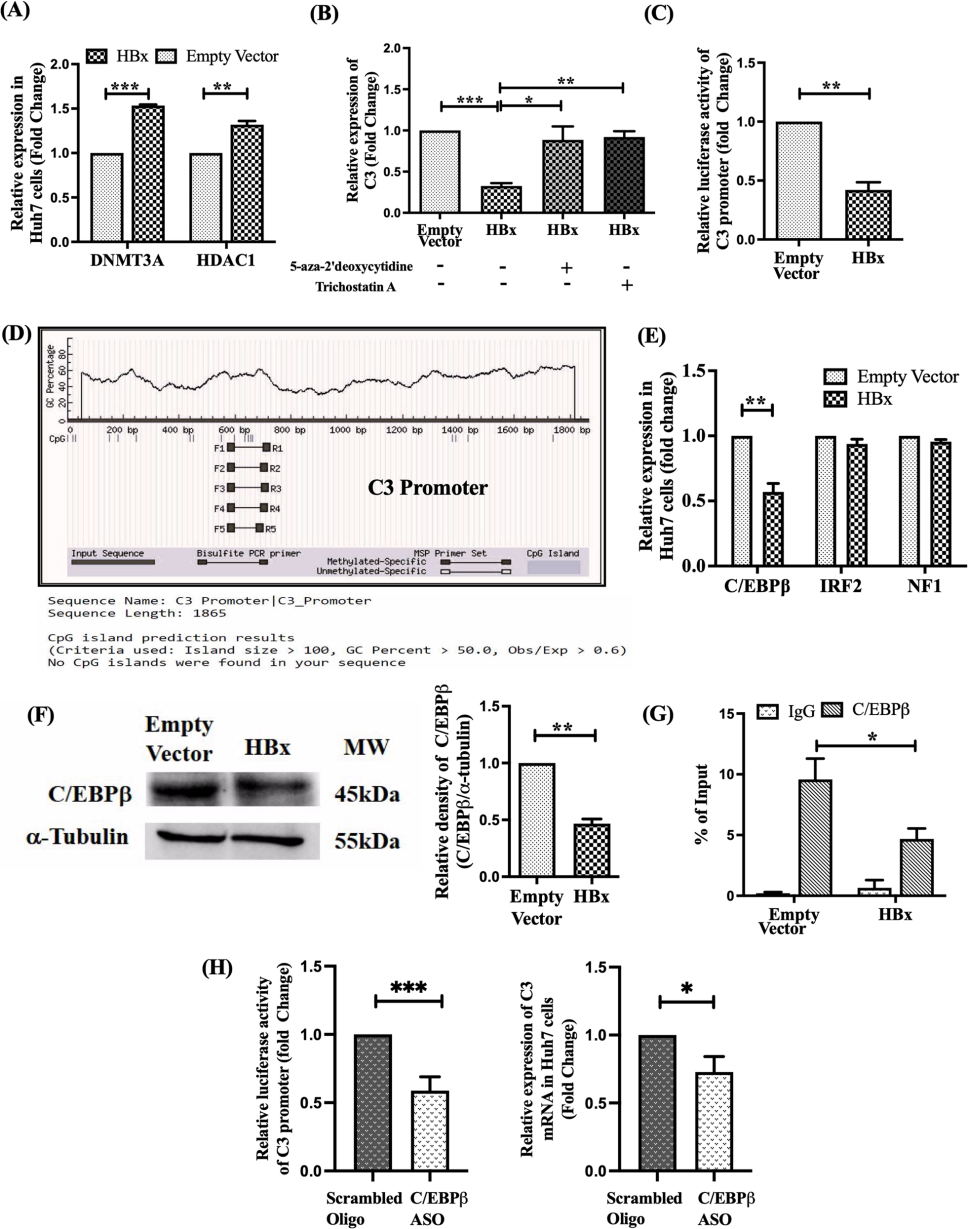
HBX-mediated suppression of C3 via epigenetic silencing and transcription factor modulation. **A** Relative mRNA expression of DNMT3A and HDAC1 in Huh7 cells transfected with HBx. **B** C3 mRNA expression in HBx-transfected Huh7 cells treated with or without DNA methylation inhibitor 5-aza-2’-deoxycytidine (5 μM) or histone deacetylation inhibitor Trichostatin A (0.1 μM). **C** Relative luciferase activity of C3 promoter-luciferase reporter construct (pGL3-C3-Prom) co-transfected with HBx or empty vector. **D** In silico prediction of CpG island within C3 promoter using online tool Methprimer. **E**, **F** Relative expression of C/EBPβ, NF1 and IRF2 mRNA (**E**) and C/EBPβ protein (**F**) in HBx- or empty vector-transfected Huh7 cells determined by real time PCR and Western blot, respectively. Cellular α-Tubulin served as the loading control for Western blot. **G** ChIP assay showing C/EBPβ occupancy at the C3 promoter in HBx- and empty vector-transfected Huh7 cells. IgG served as negative control; results were normalized to input DNA (5%). **H** Relative luciferase activity of the pGL3-C3-Prom reporter plasmid co-transfected with antisense oligo (C/EBPβ-ASO) (1200 ng/mL) or control scrambled oligo in Huh7 cells and relative C3 mRNA expression in Huh7 cells transfected with C/EBPβ-ASO or scrambled oligo. Data are shown as mean ± SD from three independent experiments. Paired *t*-tests were used for statistical comparisons. *p < 0.05, **p < 0.005, ***p < 0.0001

Transcriptional silencing of a gene may occur due to epigenetic modifications like DNA hypermethylation or histone deacetylation [19] and HBX is known to regulate the expression of a variety of host genes by inducing these epigenetic changes [20]. In order to explore HBX-dependent epigenetic alteration in the regulation of C3 expression, we first examined in HBX-expressing Huh7 cells, the expression of two important epigenetic regulators, DNA methyltransferase 3a (DNMT3A) and histone deacetylase 1 (HDAC1) that maintain DNA methylation and histone deacetylation respectively. Our results indicated that the expression of both DNMT3A and HDAC1 expression levels were markedly elevated in HBX-expressing Huh7 cells compared to the empty-vector control (Fig. 2A).

Further to test whether HBX inhibits C3 expression by induction of aberrant DNA methylation or histone deacetylation, HBX-transfected Huh7 cells were separately treated with DNMT3A inhibitor, 5-Aza-2′-deoxycytidine and HDAC inhibitor, TSA and C3 expression was analyzed. An induction of C3 mRNA was perceived by both 5-Aza-2′-deoxycytidine and TSA in HBX-transfected Huh7 cells, suggesting the involvement of both these epigenetic mechanisms in C3 downregulation (Fig. 2B).

### HBX inhibits C3 promoter activity

To further elucidate the mechanism underlying HBX-mediated suppression of C3, we first assessed the impact of HBX on C3 promoter activity. The C3 promoter was cloned upstream of the firefly luciferase gene to generate the pGL3-C3-Prom reporter construct, which was co-transfected into Huh7 cells along with pcDNA3.1/myc-His-HBx or an empty vector and the pRL-CMV plasmid expressing Renilla luciferase as an internal control. The reporter assay demonstrated that HBX expression significantly repressed C3 promoter activity, as evidenced by an approximately 2.1-fold reduction in luciferase signal compared to the empty vector control (Fig. 2C).

### Lack of CpG islands in C3 promoter

To investigate the possibility of C3 promoter inactivation via HBX-induced hypermethylation, we first attempted to locate CpG islands within the C3 promoter using MethPrimer program [18]. However, in silico analysis revealed that CpG islands are absent in the promoter of C3 gene (Fig. 2D), suggesting that HBX does not directly methylate this promoter region but may act through upstream transcription factors.

### HBX mediated alteration in the expression of the TF C/EBPβ that binds to C3 promoter

Insufficient availability of TFs binding to a promoter can lead to diminished promoter activity and reduced gene expression. Utilizing the online tools PROMO and TFBIND with a stringency threshold of < 5% dissimilarity and a sequence length of ≥ 10 nucleotides, we analyzed the C3 promoter and identified three candidate TFs: C/EBPβ, IRF2 and NF1. We subsequently evaluated the expression of these factors in HBx-transfected Huh7 cells via RT-qPCR. Our results demonstrated that C/EBPβ mRNA was significantly downregulated by approximately 1.8-fold in the presence of HBX, whereas no significant changes were observed in IRF2 or NF1 levels compared to empty-vector controls (Fig. 2E). This inhibitory effect of HBX on C/EBPβ was further confirmed at the protein level by Western blot analysis (Fig. 2F, S3). These findings suggest that the suppression of C/EBPβ expression likely contributes to its decreased binding to the C3 promoter, resulting in attenuation in transcriptional activity.

To validate the binding of C/EBPβ to the C3 promoter, a ChIP assay was performed in Huh7 cells transfected with pcDNA3.1/myc-His-HBx or an empty-vector control. Enrichment of C3 promoter DNA was observed following immunoprecipitation of the chromatin with an anti-C/EBPβ antibody, whereas no such enrichment was detected with the IgG negative control (Fig. 2G). Notably, the occupancy of C/EBPβ on the C3 promoter was significantly reduced by approximately twofold in the presence of HBX compared to the vector control (Fig. 2G). In all cases, immunoprecipitated DNA was quantified via RT-qPCR and normalized to input DNA levels.

To further confirm the functional role of C/EBPβ in regulating C3 transcription, C/EBPβ was knocked down in Huh7 cells using an antisense oligonucleotide (C/EBPβ-ASO). This resulted in a ~ 2.5-fold reduction in C3 promoter luciferase activity and ~ threefold decrease in C3 mRNA expression compared to control scrambled oligo (Fig. 2H). Collectively, these findings demonstrate that C/EBPβ positively regulates C3 transcription, while HBX suppresses C3 expression by reducing C/EBPβ availability and its binding to the C3 promoter.

### HBX hypermethylates C/EBPβ promoter and attenuates C/EBPβ and C3 transcription

**Fig. 3:**
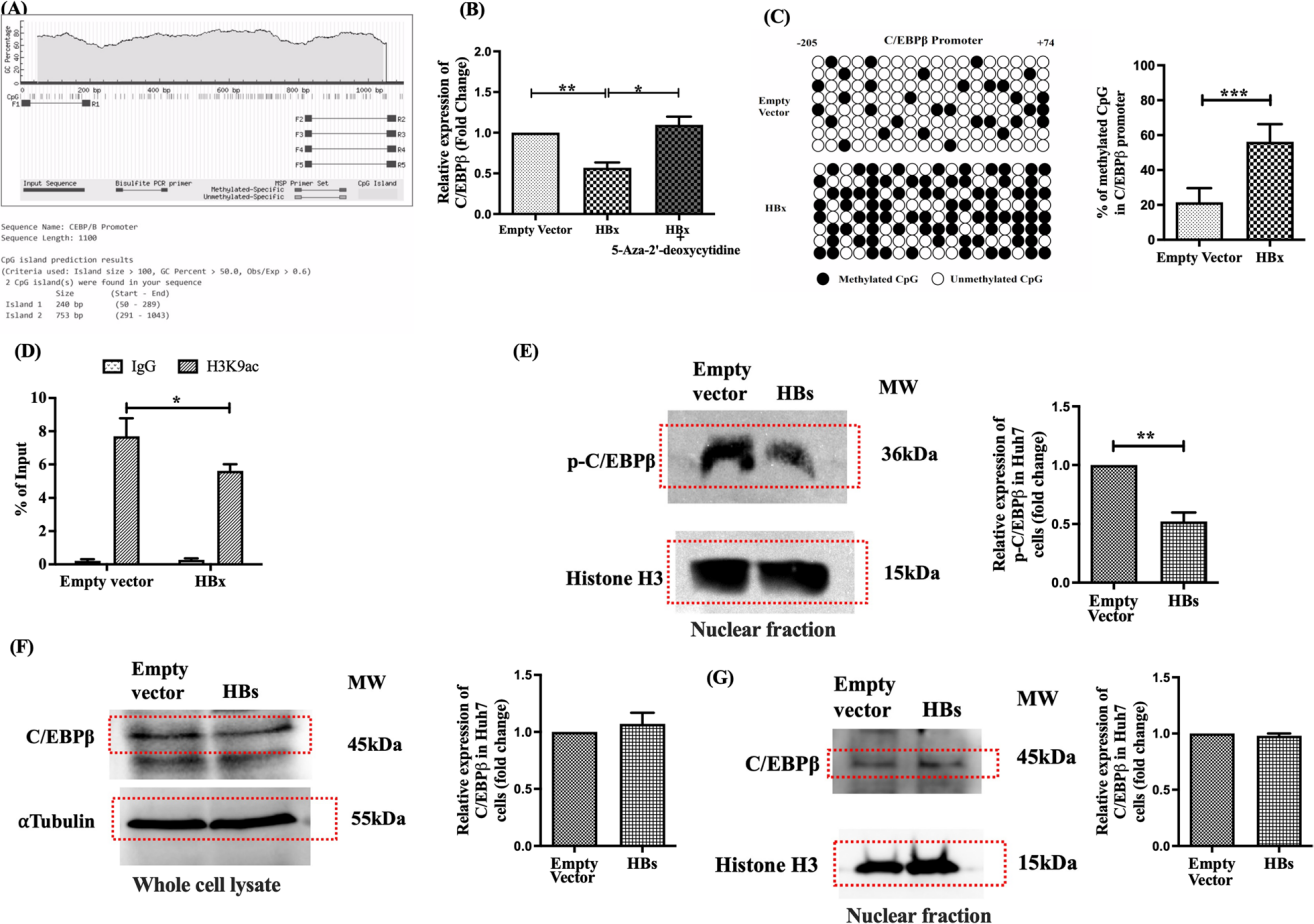
HBV proteins differentially modulate epigenetic marks and transcription factor expression impacting C3 regulation. **A** CpG island prediction in C/EBPβ promoter using Methprimer online software. **B** Relative expression of C/EBPβ in HBx transfected Huh7 cells in presence or absence of 5-aza-2’-deoxycytidine (5 μM). **C** Bisulfite sequencing analysis of CpG methylation within the C/EBPβ promoter in HBx- and vector-transfected Huh7 cells. Each horizontal line represents one clone; vertical ticks indicate CpG sites. Closed circles represent methylated, and open circles unmethylated cytosines. The percentage of methylated CpGs was calculated and compared between groups. **D** ChIP assay showing enrichment of acetylated histone H3 (H3K9Ac) at the C3 promoter in HBx- and vector-transfected Huh7 cells. IgG was used as negative control. ChIP DNA was normalized to 5% input. **E**–**G** Protein levels of phosphorylated C/EBPβ (p-C/EBPβ) in nuclear fractions (**E**), and total C/EBPβ in whole cell lysate (**F**) and nuclear fraction (**G**) from HBs-transfected Huh7 cells determined by Western blot. Histone H3 and α-Tubulin served as loading controls for nuclear proteins and proteins in whole cell lysate respectively. Mean ± SD are based on three independent experiments. Paired *t*-tests were performed for comparing the paired groups. *p < 0.05, **p < 0.005, ***p < 0.0001

The decreased binding of C/EBPβ to C3 promoter in HBX-expressing Huh7 cells could be attributed to the lower abundance of C/EBPβ in these cells and we sought to identify the molecular mechanism underlying the downregulation of C/EBPβ by HBX. Inspection of the promoter region (nt. -1000 to + 100) of C/EBPβ gene using MethPrimer indicated the presence of prominent CpG island almost throughout the promoter (Fig. 3A) and we speculated that HBX-induced hypermethylation of C/EBPβ promoter may be responsible for the deficit of C/EBPβ gene expression. To confirm this hypothesis, HBX-expressing Huh7 cells were treated with 5-Aza-2′-deoxycytidine and a marked elevation of C/EBPβ as well as C3 transcripts was observed relative to untreated cells (Figs. 3B, 2B). In addition, we evaluated by bisulfite sequencing, the DNA methylation patterns across C/EBPβ promoter in presence or absence of HBX. The frequency of C/EBPβ promoter methylation in HBx-expressing Huh7cells was found to be significantly high (~ 56.2%) than that of control cells (~ 21.5%) (Fig. 3C). Together, these data suggest that HBX induces CpG methylation at C/EBPβ promoter that results in the downregulation of C/EBPβ and restricts its binding to the C3 promoter and thereby leads to suppression of C3 expression.

### Decrease of acetylation of histone H3 in C3 promoter region in HBx-transfected Huh7 cells

Previous studies have depicted that acetylation level of H3 lysine 9 in promoter region correlates with the binding of transcription factors and gene expression [21]. Conversely, deacetylation by HDACs leads to repressed transcription [22]. As mentioned earlier, we noticed an overexpression of HDAC1 in HBX-expressing Huh7 and to establish the direct involvement of histone deacetylation at C3 promoter with low C3 mRNA expression, we performed ChIP assay using anti-acetyl-H3K9 (H3K9ac) antibody and the precipitated chromatin DNA was estimated by qPCR with primer pairs encompassing different regions of C3 promoter including C/EBPβ binding site. In all cases, the occupancy of H3K9ac on C3 promoter was found to be remarkably reduced in presence of HBX as opposed to vector-control (Fig. 3D), suggesting that decrease of H3K9ac presented a significant deacetylation of H3K9 that contributed to diminished C3 synthesis, presumably by altering the chromatin structure, making it less accessible to transcription factors and other regulatory proteins.

### HBS diminishes C3 synthesis by inhibiting C/EBPβ phosphorylation

In addition to HBX, we attempted to elucidate the mechanism by which HBS regulates the transcription of C3. Our results have highlighted that the induction of C3 transcription is facilitated by C/EBPβ. Moreover, it has been previously reported that human C/EBPβ has different phosphorylation sites, including Thr235, which are important for its transcriptional activity [23]. On the other hand, recent studies had documented that HBsAg could inhibit the phosphorylation of host proteins thereby affecting their functions [24]. We speculated that HBsAg could downregulate the transcription of C3 by blocking the phosphorylation of C/EBPβ. To test this hypothesis, the expression level of total C/EBPβ protein in both whole cell extract and nuclear extract and that of phosphorylated C/EBPβ in the nuclear extract of HBs-transfected Huh7 cells were examined through Western blot analysis using anti-C/EBP beta Antibody (H-7) and anti-phospho-Thr235 C/EBPβ-specific antibody respectively and compared with that in Huh7 cells transfected with empty-vector. As shown in Fig. 3E and S4, the phosphorylated C/EBPβ in nuclear extract was significantly reduced in Huh7-HBs cells as compared to empty-vector transfected cells although there is no variation in the total C/EBPβ protein level in both whole cell and nuclear fractions (Fig. 3F, S5; 3G, S6). Together, the results indicate that unlike HBX, HBS did not alter the synthesis of C/EBPβ or its nuclear translocation rather, it diminished its transactivation activity by suppressing the phosphorylation event, which in turn attenuate C/EBPβ-dependent transcription of C3.

### HBV mediated C3 downregulation facilitates release of virus from hepatocytes by inhibiting autophagosome-lysosome fusion

**Fig. 4:**
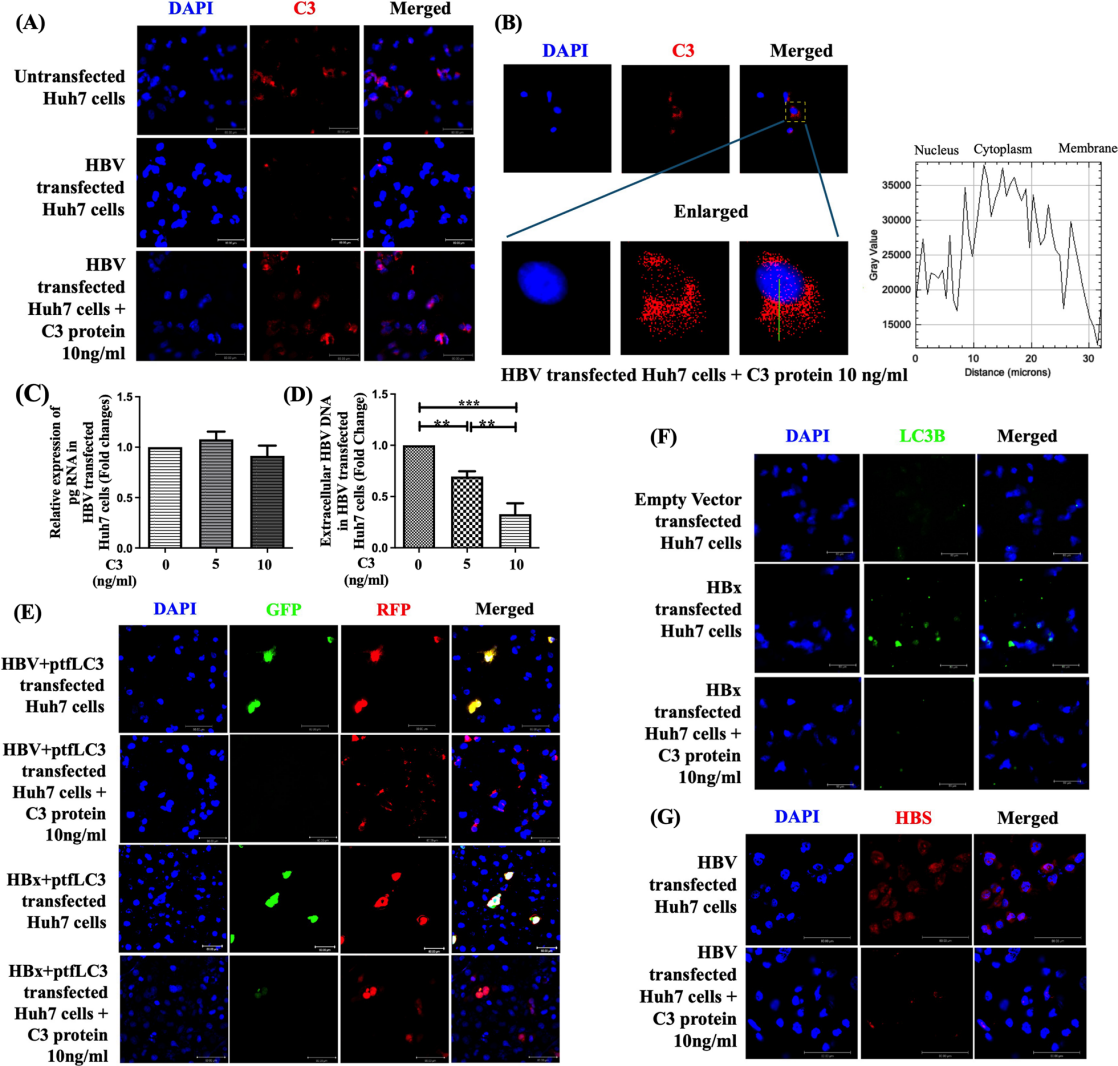
HBV reduces C3 to inhibit autophagosome-lysosome fusion and facilitate viral release. **A** Representative immunofluorescence images (40X) showing C3 (red) in full-length HBV-transfected Huh7 cells with or without recombinant C3 protein treatment (10 ng/ml) compared to untransfected cells. **B** Representative (40X z-stack) image of HBV-transfected Huh7 cells treated with recombinant C3 protein. The lower panel shows enlarged view of the yellow boxed region demonstrating cytoplasmic localization of C3. Distribution of total C3 protein was further characterized by performing a fluorescence intensity line profile, drawn from the nucleus to the plasma membrane, as indicated by the green line overlay. **C**, **D** Relative expression (fold change) of HBV pre-genomic RNA (pgRNA) (**C**) and extracellular HBV DNA (**D**) in full-length HBV-transfected Huh7 cells treated with different doses of recombinant C3 protein (0, 5, 10 ng/ml). **E** Representative immunofluorescence images at 40X magnification showing early autophagosome (both GFP and RFP-positive, green + red, yellow) and late autophagosome (RPF-positive, Red) in Huh7 cells co-transfected with full-length HBV and ptfLC3 (mRFP-GFP-LC3) or HBX-expressing plasmid and ptfLC3, both treated with or without recombinant C3 protein (10 ng/ml). **F** Representative immunofluorescence images (40X) showing accumulation of LC3B (green) in HBx-transfected Huh7 in presence or absence of C3 (10 ng/ml). **G** Intracellular HBS in HBV transfected Huh7 cells treated with or without C3 protein (10 ng/ml), visualized by immunofluorescence at 63X magnification. In panel A,B, E-G, the nuclei were counterstained with DAPI (blue). Data in C and D are presented as mean ± SD from three independent experiments. Statistical significance was assessed using one-way ANOVA followed by Tukey’s multiple comparisons test (**p < 0.005, ***p < 0.0001)

To investigate the effect of C3 downregulation on HBV production and release from hepatocytes, we first confirmed that exogenously added recombinant C3 (rC3) protein was efficiently internalized by Huh7 cells (Fig. 4A). To further determine its intracellular distribution, z-stack images were acquired and analyzed, which revealed that the internalized C3 protein was predominantly localized within the cytoplasm. Line plot analysis demonstrated a single fluorescence intensity peak in the cytoplasmic region without any secondary peaks at the cell periphery, indicating an absence of plasma membrane enrichment (Fig. 4B). Collectively, these findings confirm that exogenous C3 protein localizes mainly to the cytoplasm under the experimental conditions examined. Following confirmation of successful internalization of rC3 protein, Huh 7 cells transfected with full-length HBV were treated with different concentration of rC3 protein (5 ng/ml and 10 ng/ml). We then evaluated the levels of intracellular pregenomic-RNA (pgRNA), the major HBV transcript that serves as the template for reverse transcription-mediated synthesis of viral DNA, as well as the levels of released HBV-DNA present in the cell-culture supernatant and compared the results with those in C3-untreated control cells. While no difference in pgRNA level was detected by RT-qPCR in HBV transfected Huh7 cells with or without treatment with rC3 (Fig. 4C), HBV-DNA was found to be significantly reduced in the culture supernatant of rC3-treated Huh7 cells in dose-dependent manner compared to untreated cells (Fig. 4D). The results imply that downregulation of C3 by HBV does not affect viral transcription but favour HBV release from the hepatocytes. Previous studies have suggested that HBV induces partial autophagy to facilitate its own replication and release [25]. HBV could induce the formation of autophagosomes in hepatic cells [26] but blocks the fusion of autophagosomes with lysosomes [25]. Further, it has been documented that complement C3 is necessary for the fusion of autophagosomes and lysosomes to complete the degradation cycle [27]. We speculated that HBX by reducing C3, arrests the autophagosome-lysosomes fusion and prevents the degradation of autophagic cargo including the viral proteins or virions, resulting in greater release of HBV from the infected cells. To lend credence to this hypothesis, we transfected Huh7 cells with full-length HBV or HBX-expressing plasmid together with the tandem reporter construct, mRFP-GFP-LC3 (ptfLC3) and studied the dynamics of autophagy upon treatment with or without exogenous C3 (10 ng/ml). The ptfLC3 construct is designed such that exposure to the acidic lysosomal environment resulted in protonation of GFP chromophore and causes loss of green fluorescence while the red signal from RFP remains relatively stable, thereby allowing for differentiation between early autophagosomes (displaying both green and red; yellow) and late autolysosomes (red). As illustrated in Fig. 4E, the green punctate dots were found to be more abundant in both Huh7 cells expressing full-length HBV and HBX, but it shifted to predominantly red punctate dots upon treatment of these cells with C3, indicating that HBX inhibits autophagosome-lysosome fusion, which is triggered by the C3 deficiency it induces. Disruption of autophagosome-lysosome fusion could result in decrease in autophagic turnover and consequently lead to the accumulation of autophagosome and we studied the level of autophagosome marker, lipidated microtubule-associated protein 1 light chain 3B (LC3-II) [28] in HBX-or empty-vector transfected Huh7 cells by immunofluorescence imaging using anti-LC3-II antibody. It was observed that LC3-II protein was clearly raised in HBx-transfected Huh7 cells than in empty-vector transfected cells (Fig. 4F). However, supplementation of HBX-Huh7 cells with rC3 protein (10 ng/ml) reduced the LC3-II overload. Similarly, a greater abundance of intracellular HBS had been observed in HBV-transfected Huh7 cells, which was substantially diminished in presence of exogenous C3 (Fig. 4G). Taken together, our results highlighted that HBV-mediated C3 downregulation leads to dysfunctional autophagy, enabling viral components, including HBS to escape autophagic degradation and facilitate the assembly, envelopment and release of HBV from the cells.

### Low intracellular C3 stores in monocytes and HBV-specific T cells of chronically HBV-infected patients

**Fig. 5:**
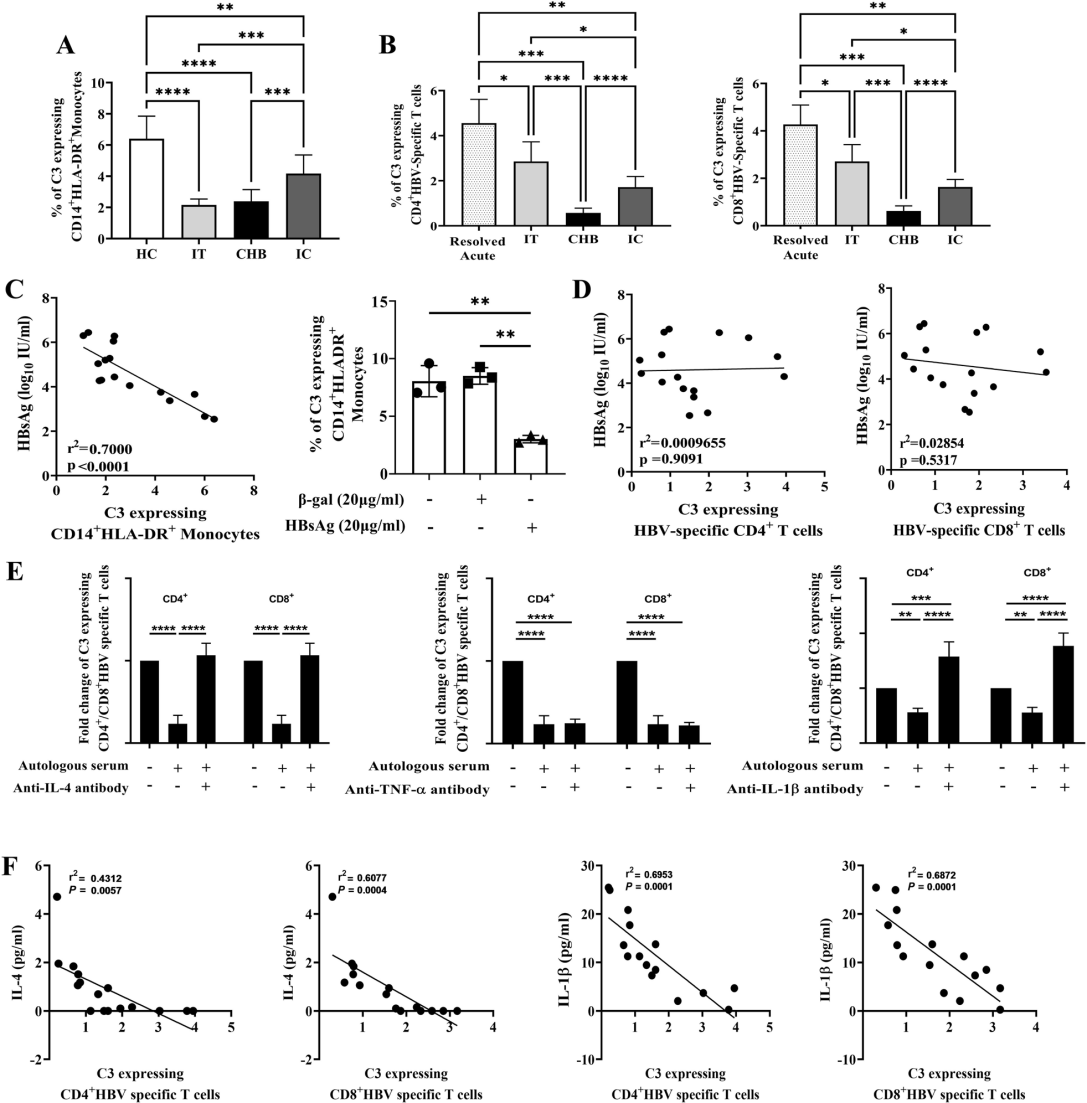
Mechanism of alteration in C3 expressing monocytes and HBV-specific T cells. **A** The percentages of C3-expressing CD14^+^HLA-DR^+^ monocytes in healthy controls (HC) (n = 20), Immune-tolerant (IT) (n = 10), chronic hepatitis B (CHB) (n = 27) and Inactive carriers (IC) (n = 22). **B** Percentages of CD4^+^ (left panel) and CD8^+^ (right panel) HBV-specific T cells expressing C3 in patients with resolved acute HBV infection (Resolved Acute) (n = 10), IT (n = 10), CHB (n = 27) and IC (n = 22). **C** Correlation analysis between percentages of C3-expressing monocytes and level of HBsAg in chronically HBV-infected patients (left panel) and the proportion of sorted CD14^+^ monocytes of HC that expressed C3 following treatment with recombinant Hepatitis B surface antigen (rHBsAg) (20 μg/ml) (right panel). Untreated cells or β-galactosidase (β-gal) (20 μg/ml) treated monocytes were kept as control. **D** Correlation analysis between percentages of C3-expressing HBV-specific CD4^+^ (left panel) and CD8^+^ T cells (right panel) with level of serum HBsAg in chronically HBV-infected patients. **E** Frequencies of C3-expressing HBV-specific CD4^+^ and CD8^+^ T cells in PBMCs of CHB treated with anti-IL-4 (left panel), anti-TNF-α (middle panel) and anti-IL-1β (right panel) neutralizing antibodies. **F** Correlation analysis between percentages of C3 expressing HBV-specific CD4^+^ and CD8^+^ T cells with level of serum IL-4 (two left panels) and IL-1β (two right panels). Statistical significance was assessed in A, B, C (right panel) and E by one way ANOVA followed by Tukey’s Multiple Comparison Test (**p < 0.005, ***p < 0.001and ****p < 0.0001). For C (left panel), D and F, statistical significance was determined by linear regression analysis

The immune cells such as, monocytes and T cells have been reported to synthesize C3, which can participate in the generation and modulation of immune responses [4]. Given that chronically HBV infected patients are characterized by impaired innate and adaptive immunity [29], we reasoned that this could be related to alteration in intracellular C3 system in the immune cells of these patients. To better understand the contribution of C3 in immune functions during CHI, we first assessed the frequency of C3-expressing HLA-DR^+^CD14^+^ total monocytes of chronically HBV infected patients in different disease phases as well as in healthy individuals by flow cytometry (Fig. S1). The frequency of intracellular C3-positive total monocytes was found to be significantly reduced in IT (2.16 ± 0.38%) and CHB patients (2.39 ± 0.75%) relative to IC (4.17 ± 1.19%) and HC (6.40 ± 1.45%) (Fig. 5A). However, C3^+^-monocytes were significantly lower in IC than HC.

We further analysed the percentage of intracellular C3-positive HBV-specific CD4^+^ and CD8^+^ T cells of IT/CHB/IC patients and compared it with that seen in acutely HBV infected patients who had resolved infection. For this, PBMCs of study subjects were stimulated with HBV-specific peptides for 5 days and IFN-γ producing CD4^+^/CD8^+^ T cells were identified as the HBV-specific T cells and intracellular C3 expression in these cells were studied (Fig. S2). In comparison to the percentages of C3-producing HBV-specific CD4^+^/CD8^+^ T cells in patients with resolved acute HBV infection (CD4^+^ T cells: 4.55 ± 1.05; CD8^+^ T cells: 4.27 ± 0.82), a marked decline in these cell proportions was noted in all chronically HBV-infected patients, although a variability in C3 level had been observed across the disease phases of CHI. The C3 deficiency was most pronounced in virus-specific CD4^+^/CD8^+^ T cells of CHB patients (0.57 ± 0.21/ 0.62 ± 0.21) followed by IC (1.71 ± 0.47/ 1.63 ± 0.31) while C3 was relatively high in IT (2.85 ± 0.87/ 2.71 ± 0.71) (Fig. 5B).

### High HBsAg level downregulates C3 expression in monocytes but not in HBV-specific T cells of CHB patients

We next sought to identify the viral antigen that might contribute to the decreased synthesis of C3 in monocytes and virus-specific T cells in CHB patients. HBsAg is the most abundant viral protein in the blood of HBV-infected individuals and we evaluated HBsAg concentration in sera of patients representing different phases of CHI. HBsAg level was found to be equivalent in IT and CHB patients but markedly low in IC (Fig. S7). A strong inverse correlation was observed between serum HBsAg titre and the frequencies of C3-expressing monocytes (Fig. 5C). Further, treatment of CD14^+^ monocytes sorted from HC with rHBsAg resulted in significant decrease in intramonocytic C3 level respectively in comparison to untreated and β-Gal treated control cells (Fig. 5C).

In contrast, no significant correlation was found between the serum HBsAg concentration and intracellular C3 level in HBV-specific CD4^+^ and CD8^+^ T cells, implying that quantitative HBsAg exerts little effect on the reduction of C3 stores in HBV-specific T cells (Fig. 5D).

### IL-4 and IL-1β attenuate intracellular C3 in HBV-specific T cells of CHB patients

The lack of proportional relationship between T-cell intrinsic C3 levels and HBsAg quantity prompted us to explore whether the systemic cytokines could lower the production of C3 within HBV-specific CD4^+^ and CD8^+^ T cells. Quantification of serum cytokines with the flow cytometric bead array depicted significant increases in the levels of TNF-α and IL-4 exclusively in CHB than other groups (IT/IC/HC), while IL-1β had significantly higher concentration in CHB and IC relative to IT or HC (Fig. S8). On the other hand, IFN-γ level remained comparable in HC/CHB/IC but low in IT. Given that the virus-specific T cells of CHB patients are characterized by marked depletion of intracellular C3 stores, we examined whether the cytokines TNF-α, IL-4 and IL-1β that are high in CHB could result in diminished C3 synthesis in these T cells. Our in vitro experiments demonstrated that PBMC of CHB patients stimulated with HBV-specific peptides and incubated with autologous serum (having high levels of TNF-α/IL-4/IL-1β) showed diminished C3 levels, while addition of anti-IL-4 or anti-IL-1β but not anti-TNF-α antibody, resulted in notable improvement in C3 expression in both HBV-specific CD4^+^ and CD8^+^ T cells (Fig. 5E). Further, a strong inverse correlation was perceived between C3-expressing HBV specific CD4/CD8^+^ T cells and serum IL-4 and IL-1β concentration (Fig. 5F), signifying the central roles of IL-4 and IL-1β in the down-regulation of C3 in virus-specific T cells.

### Low C3 in monocytes and HBV-specific T cells of CHB patients leads to aberrant cytokine responses

**Fig. 6:**
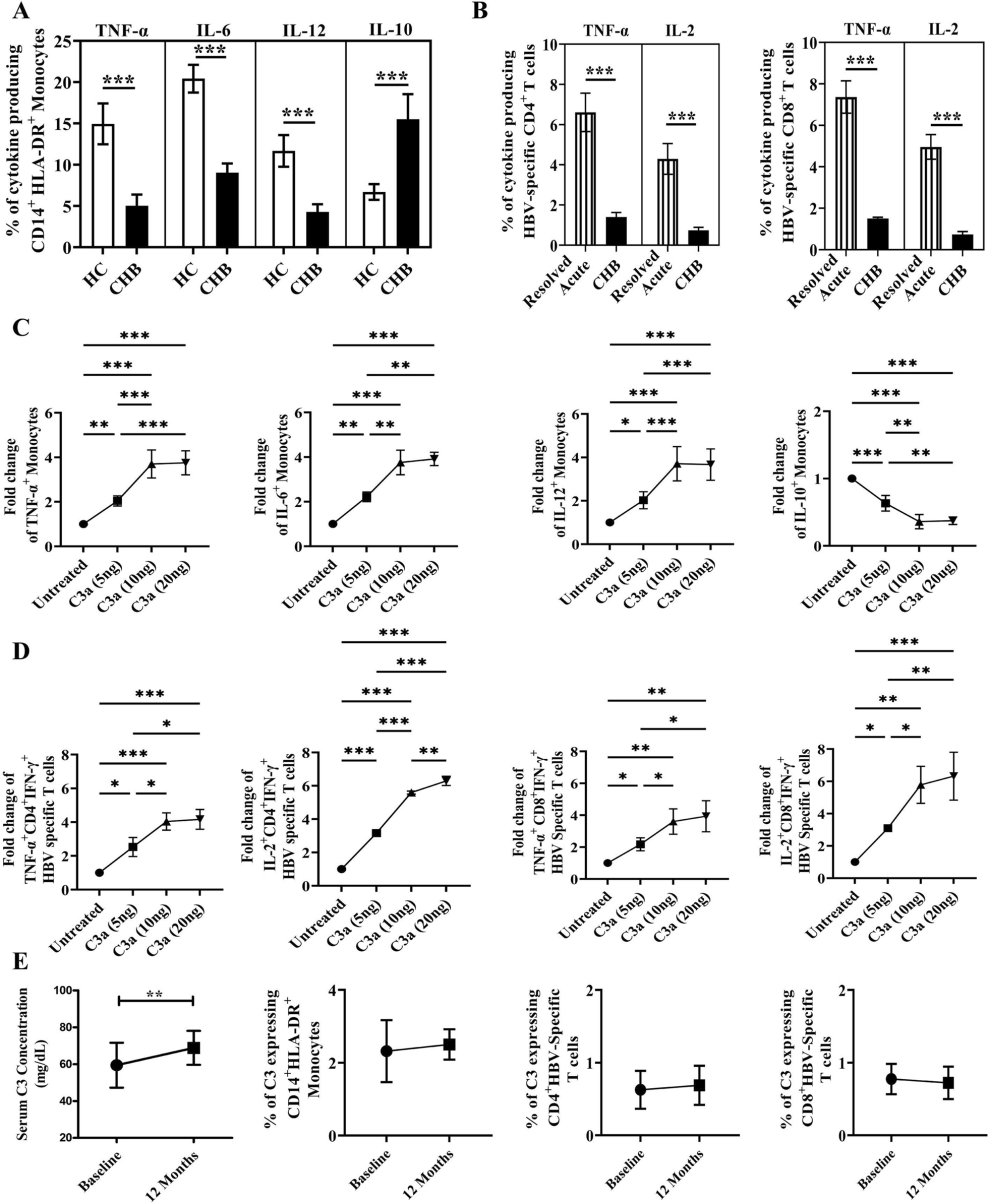
C3 restores cytokine balance in monocytes and HBV-specific T cells; C3 levels in TDF-treated patients. **A** Grouped bar diagrams demonstrating percentages of TNF-α^+^, IL-6^+^, IL-12^+^ and IL-10^+^ monocytes in healthy controls (HC) (n = 15) and chronic hepatitis B (CHB) patients (n = 15). **B** Bar diagrams depicting percentages of HBV-specific CD4^+^ (left panel) and CD8^+^ T cells (right panel) expressing cytokines TNF-α and IL-2 in acutely HBV infected patients with resolved infection (Resolved Acute) (n = 10) and CHB patients (n = 15). **C** Frequencies of TNF-α^+^, IL-6^+^, IL-12^+^ and IL-10^+^ monocytes in PBMCs of CHB patients treated with recombinant C3a in different concentrations (5, 10, 20 ng/ml). **D** Percentages of HBV specific CD4^+^ and CD8^+^ T cells expressing cytokines TNF-α and IL-2 in HBV peptide stimulated PBMCs of CHB patients treated with recombinant C3a in different concentrations (5, 10, 20 ng/ml). **E** Serum concentration of C3 and frequency of C3 expressing monocytes and HBV specific CD4^+^ and CD8^+^ T cells in CHB patients (n = 10) before initiation (Baseline) and after oe year of antiviral Tenofovir therapy (12 Months). In panels A, B and E *t*-tests were performed for comparing groups (**p < 0.01, **p < 0.001). For C and D, comparisons between groups were performed using the one-way ANOVA with *p* values adjusted by the Tukey’s multiple comparison test (*p < 0.05, **p < 0.01, ***p < 0.001, ****p < 0.0001)

Previous research had demonstrated impaired effector functions of monocytes and HBV-specific T cells in CHB patients [30, 31]. To explore whether decrease in intracellular C3 in these cells contribute to this defect, we first studied the cytokine production by monocytes and HBV-specific CD4^+^-/CD8^+^- T cells in CHB patients. We observed a marked decline in the production of proinflammatory cytokines TNF-α, IL-6, IL-12 and enhanced expression of inhibitory cytokine IL-10 by monocytes derived from C3-deficient CHB patients as compared with HC (Fig. 6A). In parallel, low frequencies of TNF-α- and IL-2-producing HBV-specific CD4^+^-/CD8^+^-T cells were also noted in CHB while such cells were markedly high in patients with resolved HBV infection (Fig. 6B). Separate sets of experiments were performed to compare the cytokine expression by monocytes and HBV-specific T cells in the presence of different concentration of rC3a relative to untreated cells. The flow cytometric data indicated that there was gradual increase in the percentages of TNF-α-, IL-6- and IL-12- expressing monocytes and a decrease in IL-10^+^-monocytes in CHB patients upon addition of increasing doses (5 ng/ml and 10 ng/ml) of C3a, with gradual saturation at higher dose of 20 ng/ml (Fig. 6C). Similarly, a marked improvement in the abilities of HBV-specific CD4^+^ and CD8^+^ T cells to produce TNF-α and IL-2 was perceived upon addition of C3a in dose dependent manner as compared to the untreated cells (Fig. 6D), reinforcing the critical role of C3 in immune cell functions.

### Tenofovir therapy partially restores serum C3 but not intracellular C3 in immune cells

Tenofovir is recommended as first-line monotherapy for patients with CHB [32] and we tested the effect of one year of Tenofovir treatment on the C3 levels in sera and within monocytes and HBV specific T cells in 10 CHB patients. We observed that all patients achieved < 250 copies/ml of HBV DNA and normalization of serum ALT after 1 year of therapy (Fig. S9). There was a slight increase in the serum C3 level after therapy, however they remained substantially lower than that observed in healthy individuals. Further, no significant change was perceived in proportion of C3-expressing monocytes or HBV-specific T cells between pre-treatment and post-treatment time points (Fig. 6E). In addition, the serum levels of HBsAg, IL-4 and IL-1β in these patients remained similar to the baseline values following 1 year of treatment (Fig. S10).

## Discussion

The identification of specific mechanisms employed by viruses to evade the complement system provides the most compelling evidence that complement plays a significant role in the host defence against virus and influence the outcome of infection. The hepatocytes of the liver are the principal site of HBV replication [32] and are also primarily responsible for the biosynthesis of about 80–90% of plasma complement components, including C3 [1]. Moreover, C3 is also produced locally by immune cells such as T cells and monocytes and plays an important role in driving the immune response [33]. In the present study we uncovered the mechanism by which HBV proteins, HBX and HBS along with the systemic cytokines (IL-4 and IL-1β) suppress the production of C3 by hepatocytes and immune cells during CHI and contribute to disease progression by promoting increased viral production at one hand and attenuating the anti-viral immune response at the other hand.

To gain an insight into the effect of HBV on C3, we studied C3 expression in HBV transfected/infected human hepatoma cells and also in sera, liver tissue sections and immune cells of chronically HBV infected patients. In all cases, a marked reduction in C3 expression was perceived relative to controls. Notably, hepatic C3 mRNA expression exhibited a more pronounced reduction than the corresponding decrease in circulating C3 protein levels in CHB patients. This apparent discrepancy likely reflects the fact that hepatic mRNA levels represent real-time transcriptional activity, which is highly sensitive to HBV-associated inflammatory and regulatory signals, whereas serum C3 protein indicates cumulative secretion and systemic stability with a relatively longer circulating half-life. Moreover, although hepatocytes constitute the major source of circulating C3, extrahepatic production by immune cells may also contribute to maintaining detectable serum C3 levels. Consequently, marked suppression at the transcriptional level may not translate proportionally into an equivalent immediate decrease in circulating protein abundance.

In addition, transfection experiments with a full-length linear HBV monomer or individual viral protein expression vectors revealed that, similar to full-length HBV, HBX and HBS markedly repressed C3 expression at both mRNA and protein levels, whereas HBV core and polymerase had negligible effects. To confirm the specificity of these findings, we excluded the possibility of unintended (“leaky”) HBS expression from the HBV polymerase construct, which may arise because of overlapping HBV reading frames. HBsAg secretion, assessed by ELISA (data not shown), was readily detected in cell culture media of HBs-transfected Huh7 cells but remained at background levels in HBV-P-transfected cells. These results endorsed that the HBV polymerase construct functions independently of surface protein expression, indicating that the observed modulation of C3 is specifically attributable to the intended viral proteins. Although our results clearly demonstrated reduced C3 abundance in HBX- and HBS-expressing hepatocytes, it remains unclear whether these viral proteins also influence the intracellular localization of C3, which could potentially affect intracellular complement processing and downstream signalling. These mechanistic aspects warrant further investigation.

It has been documented that HBX can transactivate or transrepress viral and cellular genes by either directly interacting with various TFs or can alter the transcription of target genes through epigenetic modifications [20]. It was noted that treatment of HBx-transfected cells with TSA as well as 5-Aza-2’-deoxycytidine could restore C3 expression, suggesting that HBX facilitates DNA methylation and histone deacetylation dependent transcriptional silencing of C3. This was further supported by the fact that HBX induced the overexpression of the enzymes DNMT3A and HDAC1 that regulate these two epigenetic modifications respectively.

To establish the involvement of HBX mediated hypermethylation events for failure to transcribe the downstream gene, we searched for CpG islands in C3 promoter. However, no CpG island could be detected in C3 promoter. This prompted us to explore the alternative possibility that changes in the availability or activity of TFs binding to C3 promoter may be related to the decrease in C3 promoter activity and C3 expression in HBX-transfected cells. By bioinformatic analysis, we identified C/EBPβ, a classic basic leucine-zipper family transcription factor to be the key regulator of C3 gene expression and a striking reduction was perceived in the abundance of endogenous C/EBPβ in HBX-expressing Huh7 cells. This implies that in presence of HBV, the trend of C/EBPβ expression was consistent with that of C3. Previous studies had implicated C/EBPβ in the induction of proinflammatory cytokines [34] and its C-terminus region contains a basic DNA binding domain that mediates its binding to the corresponding DNA binding sequences ^A^/_G_TTGCG^C^/_T_AA^C^/_T_) [35]. By ChIP-PCR assay, we demonstrated a strong binding of C/EBPβ to the C3-promoter and this was markedly decreased in presence of HBX. Moreover, it was noted that treatment with 5-Aza-2’-deoxycytidine could result in modest enhancement of C/EBPβ transcription in HBX-transfected cells, implying that HBX-induced DNA hypermethylation is associated with transcriptional repression of C/EBPβ with accompanying decrease in C3 expression. In addition, localized CpG repeat sequences were detected in the endogenous promoter region of C/EBPβ gene while bisulfite sequencing revealed extensive methylation of these CpG sites in HBx-transfected Huh7 cells. These results underscore the notion that the major decrease in C3 during CHI was due to downregulation in the expression of the transcription factor C/EBPβ, achieved through HBX-induced aberrant C/EBPβ-promoter methylation that led to prominently decreased binding of C/EBPβ to the C3-promoter, thereby diminishing C3 expression.

Further, histone deacetylation also plays a crucial role in gene silencing. Studies had shown that acetylation on histone H3 lysine 9 on specific gene promoters is linked to active transcription as it facilitates the release of RNA polymerase II (Pol II) from a paused state, allowing the transition from transcription initiation to elongation [21]. Further, C/EBPβ is known to contribute to transcriptional elongation through the recruitment and stabilization of the positive transcriptional elongation factor to the promoter [36]. Therefore, low H3K9 acetylation would likely enhance Pol II pausing on gene promoters and consequently hinder C/EBPβ-mediated transcription. We noted that H3K9 acetylation state over C3 promoter, including C/EBPβ binding site, was distinctly reduced in presence of HBX and it negatively correlated with C3 transcription. Moreover, a number of reports have suggested that the transcriptional activity of C/EBPβ, including its binding to target gene promoters is regulated by posttranslational modifications such as, phosphorylation [37, 38]. Different residues in C/EBPβ protein can be phosphorylated by multiple signalling pathways, out of which Thr-235 is the most common site for phosphorylation primarily by MAPK kinases [23]. We demonstrated that HBS inhibited Thr-235 phosphorylation in C/EBPβ protein and thus further disrupt C/EBPβ’s ability to activate C3 transcription. Collectively, the synergistic effect of HBX-induced C3-promoter deacetylation and methylation-dependent loss of C/EBPβ expression along with HBS-mediated inhibition of C/EBPβ phosphorylation resulted in transcriptional repression of C3. We further noted that this downregulation of C3 in the hepatocytes by HBV has a critical impact on viral life cycle as it significantly enhanced the release of progeny HBV particles from cells. HBV is known to activate the early autophagic pathways [26] and understanding the interplay between autophagy and HBV replication and release has been the subject of intense investigation in the past years [25]. We found that HBV, by restricting C3 availability, inhibited the fusion of autophagosomes with lysosomes and evaded autophagic degradation of viral proteins such as HBS, thereby promoting the envelopment and formation of mature viral particles. These findings thus provide a novel insight into a previously unappreciated mechanism by which HBV escapes autophagy-mediated antiviral immunity by targeting C3. Although the present work focused on the impact of C3 on HBS as a clinically relevant marker of viral output, it will be important to determine whether C3 exerts broader regulatory effects on additional HBV proteins, including HBX and HBc. Emerging evidence further suggests that intracellular cleavage of C3 can generate bioactive fragments such as C3a, capable of regulating cellular metabolism and autophagy [39]. While our study supports a predominant role for total C3 in modulating viral persistence in hepatocytes, intracellularly generated C3a may represent an additional downstream regulatory layer in HBV pathogenesis that merits future investigation.

Recent studies indicate that in immune cells, the intracellular repertoire of C3 plays a key role in regulating the effector functions and as marked impairment in host immune responses had been reported in chronically HBV infected patients, we investigated whether CHI leads to C3 deficiency in immune cells and if this lack of C3 is linked to immune dysfunctions in HBV infected patients. We observed a heterogeneity in the percentages of C3-expressing monocytes and HBV-specific T cells in different clinical stages of CHI. The monocytes of both IT ad CHB manifested a low expression of intracellular C3 than IC and HC. However, C3 was found to be significantly reduced in HBV-specific CD4^+^/CD8^+^ T cells of CHB and IC in comparison to IT. We established a causal influence of high HBsAg concentration on low C3 expression by monocytes and this concurred with decreased frequency of C3-expressing monocytes in IT and CHB patients, who unlike IC, carried high serum HBsAg levels. The circulating monocytes had been found to harbour a detectable reservoir of HBsAg in CHB patients [30]. Moreover, the sialyl glycans on HBsAg had been shown to bind to the immune checkpoint receptor Siglec-3 (CD33) to modulate host immunity [40]. We had earlier reported a heightened expression of CD33 on the monocytes of chronically HBV-infected patients compared to healthy controls [30] and it appears that HBV surface proteins exert their effects on monocytes via interaction with CD33 and similar to that seen in hepatoma cells, would affect C3 expression by altering the phosphorylation of C/EBPβ. In contrast, there was no correlation between HBsAg titre and C3-expressing HBV-specific T cells. Instead, we demonstrated that elevated levels of cytokines IL-4 and IL-1β could individually contribute to the diminished C3 levels in HBV-specific CD8^+^/CD4^+^ T cells. While the CHB patients were characterized by high levels of both IL-4 and IL-1β relative to HC/IT, the patients in IC phase elaborated a high concentration of IL-1β, similar to CHB and thus could explain the strong suppression of C3 in HBV-specific T cells seen in CHB and IC patients but not in IT. Further, our co-culture assays using exogenous C3 supplementation revealed that C3 deficiency was intimately involved in undermining the monocytes and HBV-specific T-/B-cell functions in CHI. Downregulation of C3 in monocytes of HBV infected patients has been found to cause a marked decline in the production of proinflammatory cytokines, TNF-α, IL-6 and IL-12 along with increase in anti-inflammatory IL-10 expression by these cells, whereas C3-deficient HBV-specific T cells were found to be severely impaired in producing TNF-α and IL-2, thereby inhibiting the clearance of the virus. However, in both cases these diminished effector activities could be rescued proportionally by additional C3 provision. These findings align with the observations by Kolev *et al.*, where C3 insufficiency in monocytes and T cells of patients with leukocyte adhesion deficiency-1 similarly resulted in blunted effector functions, including reduced IL-1β from monocytes and IFN-γ from CD8 + T-cells [5].

Finally, we observed that one year of Tenofovir treatment resulted in marked reduction in HBV-DNA but only a partial improvement in serum C3 concentration, while the monocytes and HBV-specific T cells continued to maintain low level of C3 similar to pre-treatment values. This limited recovery could be attributed to the inability of Tenofovir to effectively reduce the levels of HBV surface proteins, which are produced and released in several folds excess to full virions as well as to the persistence of cytokines such as IL-4 and IL-1β that suppress C3 expression. The sustained impairment of immune functions and the continued skewing towards an immunosuppressive state, despite antiviral therapy, may therefore represent a pivotal risk factor for the progression of CHI to advanced liver disease, including hepatocellular carcinoma.

## Conclusion

This study has identified novel mechanisms of complement C3 regulation during CHI leading to increased viral release and a weakened immune response against the virus. These findings would contribute to the development of novel therapeutic approaches and vaccines for restoring complement activities in chronically HBV-infected patients, thereby facilitating effective HBV control and disease management.

## Supplementary Information

Additional file1 (DOCX 1935 KB)

## Data Availability

The datasets used and/or analysed during the current study are available from the corresponding author on reasonable request.

## Abbreviations

C3: Complement C3
CHB: Chronic hepatitis B
CHI: Chronic HBV Infection
CS: Complement System
C/EBPβ: CCAAT/enhancer-binding protein beta
DNMT3A: DNA methyltransferase 3A
HBc: HBV core protein
HBeAg: Hepatitis B e antigen
HBS: HBV surface proteins
HBsAg: Small surface protein
HBV: Hepatitis B virus
HBX: HBV X protein
HC: Healthy control
HCC: Hepatocellular carcinoma
HCV: Hepatitis C virus
HDAC1: Histone deacetylase 1
IC: Inactive carrier
IT: Immune tolerant
ORF: Open reading frame
pgRNA: Pregenomic-RNA
Pol II: RNA polymerase II
r: Recombinant
SD: Standard deviation
TF: Transcription factor
TSA: Trichostatin A
ANOVA: Analysis of variance
