# Supplementary material for: Mechanistic insight into complement C3 regulation during chronic HBV infection: effect on viral persistence and host immune response
Source: J Biomed Sci. 2026 May 25;33:54. doi: 10.1186/s12929-026-01259-6 (PMC13200380; doi:10.1186/s12929-026-01259-6)
Supplement: Supplementary file 1 — Additional file1 (DOCX 1935 KB) [file 12929_2026_1259_MOESM1_ESM.docx]

**Supplementary File**

**Mechanistic insight into complement C3 regulation during chronic HBV infection: effect on viral persistence and host immune response**

*Ayana Baidya*^1^*#, Debangana Dey*^1^*#, Shreya Mallik*^1^*, Sudeshna Halder*^1^*, Najma Khatun*^1^*, Rambha Jha*^1^*, Sarthak Nandi*^1^*, Bidhan Chandra Chakraborty*^2^, *Amrita Dutta^1^, Soma Banerjee*^1^*, Abhijit Chowdhury*^3^*, SK Mahiuddin Ahammed*^3^, *Simanti Datta*^1*^

#These authors contributed equally to this work

^1^Centre for Liver Research, School of Digestive and Liver Diseases, Institute of Post Graduate Medical Education and Research, Kolkata, India

^2^Multidisciplinary Research Unit, Institute of Post Graduate Medical Education and Research, Kolkata, India

^3^Department of Hepatology, School of Digestive and Liver Diseases, Institute of Post Graduate Medical Education and Research, Kolkata, India

*Corresponding author: Prof. Simanti Datta,

Centre for Liver Research,

School of Digestive and Liver Diseases,

Institute of Post Graduate Medical Education and Research (I.P.G.M.E. & R.),

244, A.J. C. Bose Road,

Kolkata-700020, INDIA.

**Table S1:** List of primers for amplification of HBV DNA and studying host gene expression by Real-time PCR and cloning of promoter regions, bisulfite sequencing, ChIP assay

| **Primer Name** | **Primer Sequences** | **Purpose** |
| --- | --- | --- |
| F5 (Sense) | 5’ -GATGTGTCTGCGGCGTTTTA- 3’ | Quantification of HBV DNA |
| R4 (Antisense) | 5’ -AGAGGACAAACGGGCAACA- 3’ |  |
| PreG F (Sense) | 5’ CACCTCTGCCTAATCATC 3’ | Amplification of HBV pgRNA |
| PreG R (Antisense) | 5’ GGAAAGAAGTCAGAAGGCAA 3’ |  |
| C3F (sense) | 5’ GAGCCAGGAGTGGACTATGTGTA 3’ | Measurement of C3 mRNA expression |
| C3R (antisense) | 5’ CAATGGCCATGATGTACTCG 3’ |  |
| C3-prom_F (sense) | 5’-TACTGGTACCTGGTGTGAAGCCACAGATGTCAG-3’ | Cloning of C3 promoter |
| C3-prom_R (antisense) | 5’-TGTACTCGAGGTCATAACCACTCACATGGGACTC–3’ |  |
| C3-prom_ChIP_F (sense) | 5’-TGGACACAGCGGCTCACG-3’ | Amplification of C3 promoter after ChIP assay |
| C3-prom_ChIP_R (antisense) | 5’- GTGGACAAGGGGTTTCAC–3’ |  |
| C3-H3K9_ChIP_F  (sense) | 5’CAGTGAGCTGAGATAGC 3’ |  |
| C3-H3K9_ChIP_R  (antisense) | 5’GGTTACAGGCCTCTTG 3’ |  |
| C3-prom_ChIP_F(2)  (sense) | 5’-GAGGCAGTTCTTTGCTCAC-3’ |  |
| C3-prom_ChIP_R(2)  (antisense) | 5’-TGTGCCCTGTGGACAGCAC-3’ |  |
| C/EBPβ F (sense) | 5’-ACGGACACCTTCGAGGCGGC-3’ | Measurement of C/EBPβ mRNA expression |
| C/EBPβ R (antisense) | 5’-CGTAGTCGTCGGAGAAGAGG-3’ |  |
| ATF2_F (sense) | 5’ GATGAGAAGGAAGTACCATTG 3’ | Measurement of ATF2 mRNA expression |
| ATF2_R (antisense) | 5’ CTGCTGAATAATTACACTTG 3’ |  |
| IRF2_F (sense) | 5’ AAGCACATCAAGCAAGAAC 3’ | Measurement of IRF2 mRNA expression |
| IRF2_R (antisense) | 5’ CTATGATGTTCACCGTACTATC 3’ |  |
| NF1_F (sense) | 5’ GTCTTGCTGGGCAACCAAAG 3’ | Measurement of NF1 mRNA expression |
| NF1_R (antisense) | 5’ GAAGTTCAGCTGCATGCTG 3’ |  |
| 18s_F (sense) | 5’ GTAACCCGTTGAACCCCATT 3’ | Measurement of 18s rRNA expression |
| 18s_R (antisense) | 5’ CCATCCAATCGGTAGTAGCG 3’ |  |
| DNMT3A_F (sense) | 5’ CAAGAGCCCAGCACCACG 3’ | Measurement of DNMT3A mRNA expression |
| DNMT3A_R (antisense) | 5’ GCCATGGTGGGGACTTG 3’ |  |
| HDAC1_F (sense) | 5’ TGAGAACCTTAGAATGCTGC 3’ | Measurement of HDAC1 mRNA expression |
| HDAC1_R (antisense) | 5’ AGAGGGCAGATCGAGATG 3’ |  |
| CEBPB_P_BS-F  (sense) | 5’-TAGYGAGTTAGAGTYGYGT-3’ | Bisulfite sequencing of C/EBPβ promoter |
| CEBPB_P_BS-R  (antisense) | 5’- TTTATGGATTTAAAGGYA-3’ |  |
| CEBPB-ASO | 5’-GTCCCAGGCCACCAGGCGTTGCAT-3’ | Anti-sense oligonucleotide against C/EBPβ |

**Table S2:** Clinical, demographic and biochemical data of the study subjects

|  | **HC (n=20)** | **IT (n=10)** | **CHB (n=27)** | **IC (n=22)** |
| --- | --- | --- | --- | --- |
| **Age (Years),**  **Median (Range)** | 31 (25-46) | 12 (7-18) | 28 (18-58) | 33 (24-50) |
| **Sex (Male:Female)** | 13:7 | 7:3 | 20:7 | 16:6 |
| **ALT (IU/L),**  **Median (Range)** | 20 (16-25) | 30 (17-34) | 72 (48-210) | 25 (17-38) |
| **AST (IU/L),**  **Median (Range)** | 21 (17-33) | 31 (11-39) | 60 (40-174) | 25 (22-40) |
| **HBeAg status** | - | Positive | 15 Positive  12 Negative | Negative |
| **HBV DNA**  **(copies/mL)**  **Median (Range)** | - | 4.6X10^8^  (2.7X10^7-^2.3X10^9^) | 1.2X10^6^  (1.4X10^4^-4.1X10^7^) | 250  (250-4.8X10^3^) |

IT, Immunotolerant; CHB, chronic hepatitis B; IC, Inactive carriers; HC, Healthy controls; Acute resolved,; ALT, alanine aminotransferase; AST, aspartate aminotransferase; IU, international unit.

**Fig. S1**


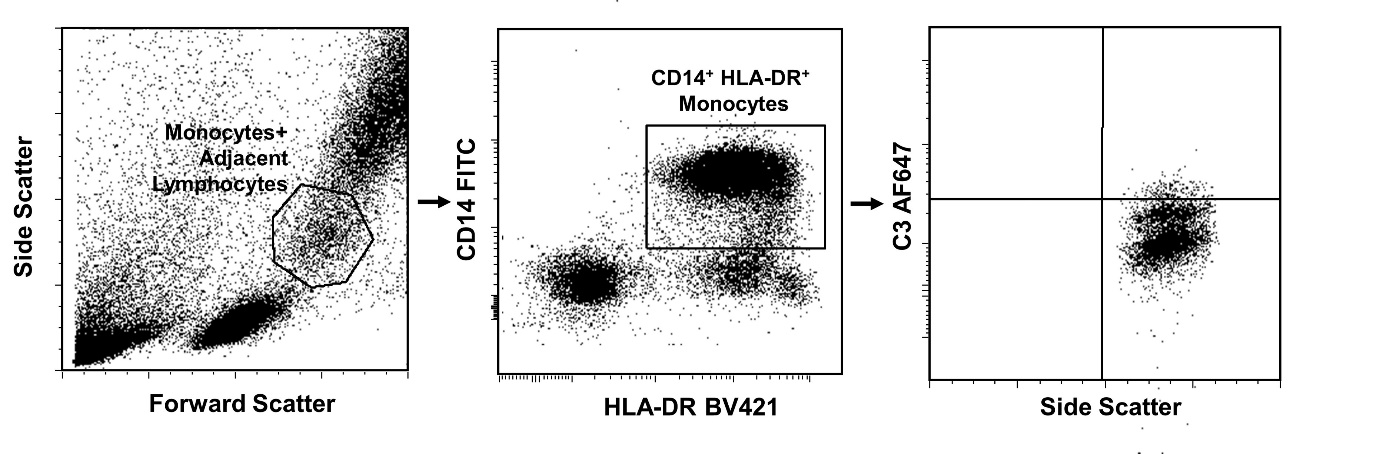


**Fig. S1.** Sequential gating strategy for identification of C3 expressing CD14^+^HLA-DR^+^ monocytes.

**Fig. S2**


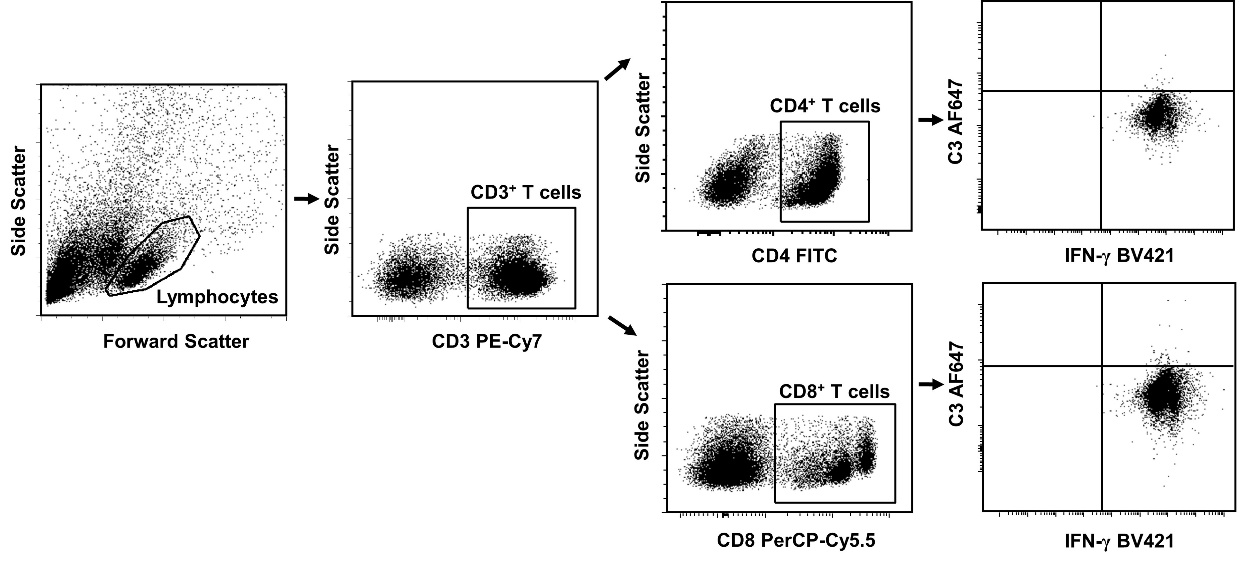


**Fig. S2.** Sequential gating strategy of IFN-γ^+^C3^+^CD4^+^/CD8^+^ HBV-specific T-cells.

**Fig. S3**


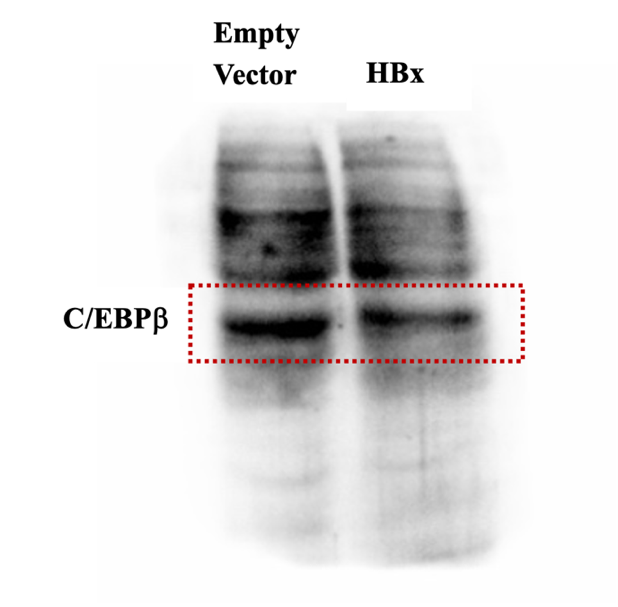

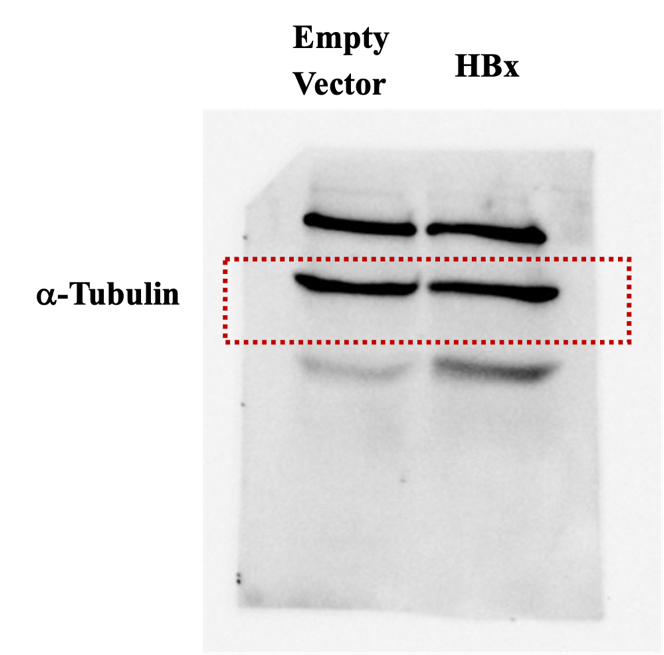


**Fig. S3.** Full-length blot showing relative expression of C/EBPβ and cellular α-Tubulin in HBx- or empty vector-transfected Huh7 cells as determined by Western blot.

**Fig. S4**

**
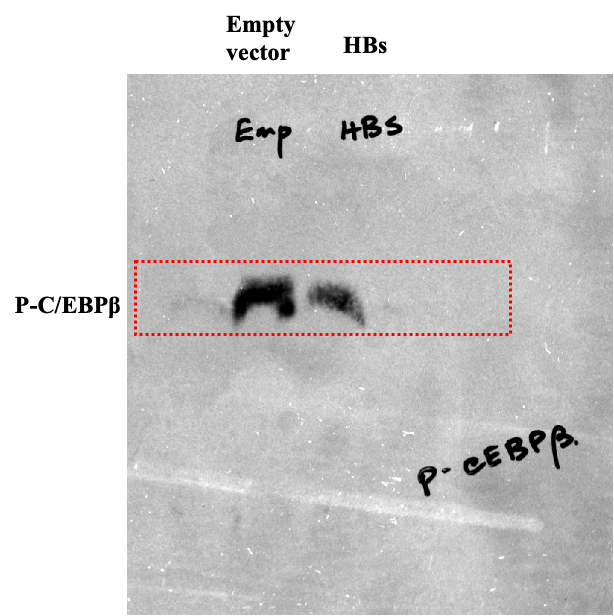

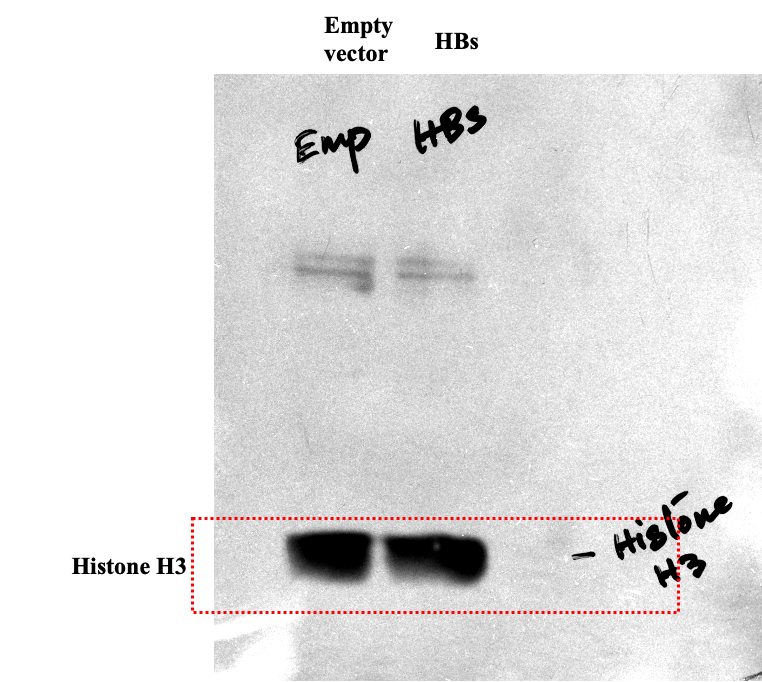
**

**Fig. S4.** Full-length blot indicating protein levels of phosphorylated C/EBPβ (p-C/EBPβ) and Histone H3 (loading control) in nuclear fractions of HBs-transfected Huh7 cells.

**Fig. S5**

**
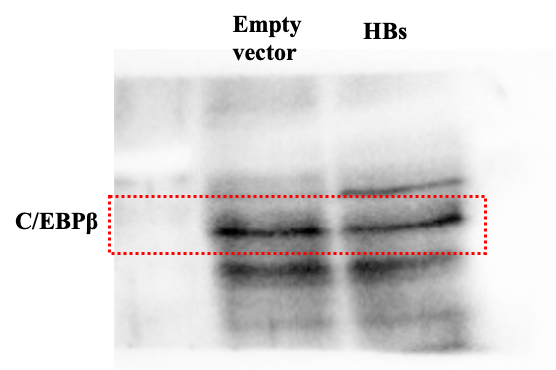

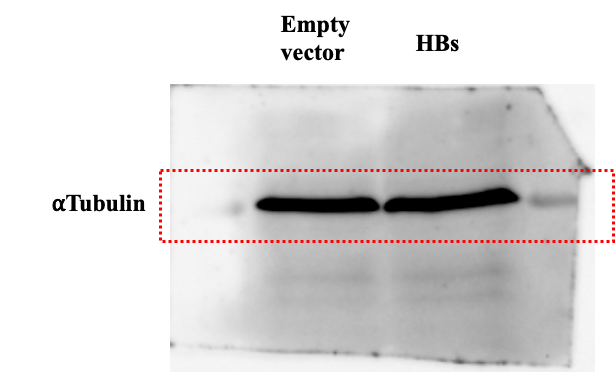
**

**Fig. S5.** Full-length blot depicting total C/EBPβ from whole cell lysate of HBs-transfected Huh7 cells and α-Tubulin which served as loading control.

**Fig.S6**

**
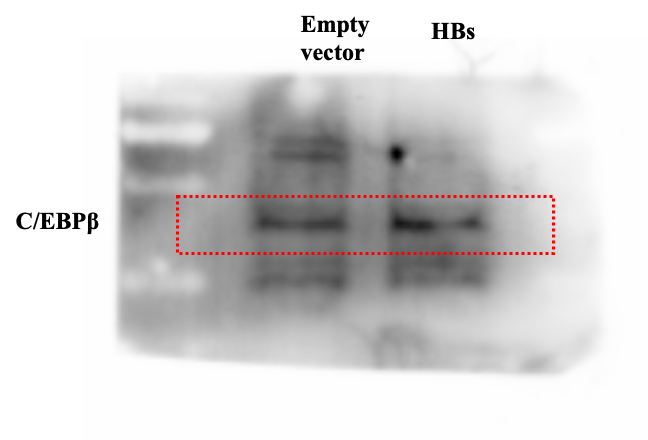

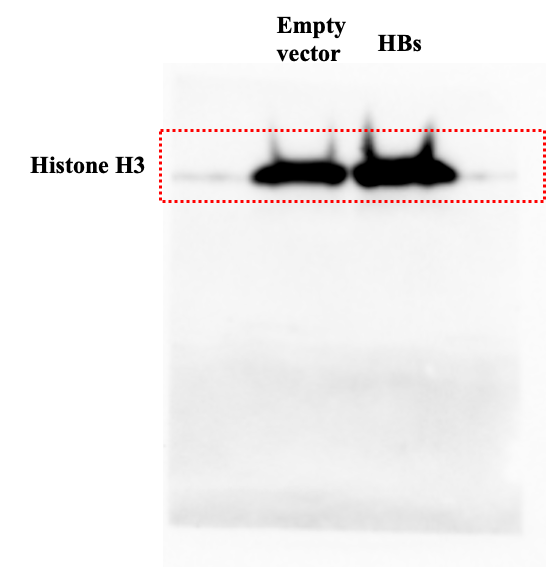
**

**Fig. S6.** Full-length blot showing total C/EBPβ from nuclear fraction of HBs-transfected Huh7 cells and Histone H3 which served as loading control.

**Fig. S7**

**
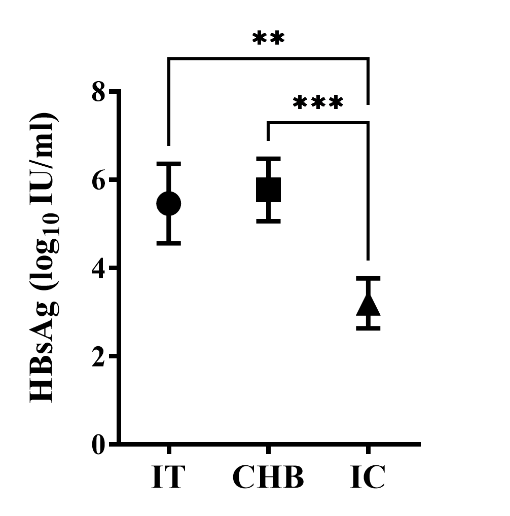
**

**Fig. S7.** Serum level of hepatitis B surface antigen (HBsAg) in Immune tolerant (IT), chronic hepatitis B (CHB) and Inactive carriers (IC).

**Fig. S8**

**
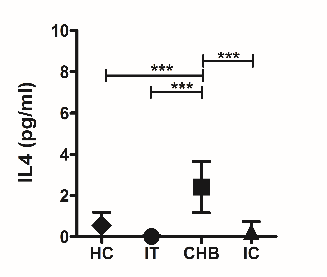

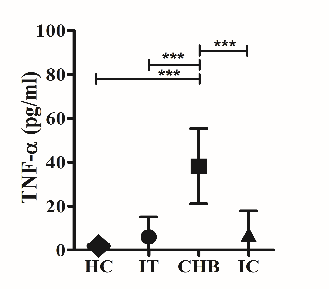
**
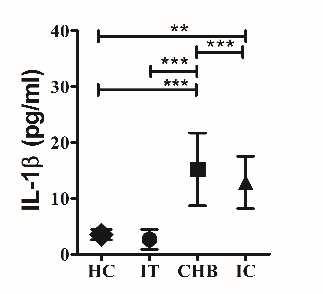

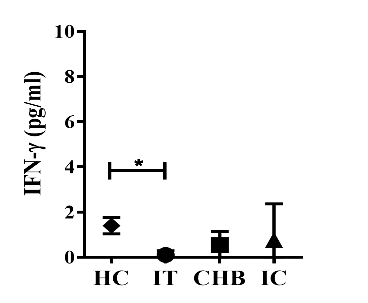


**Fig. S8.** Serum concentration of IL-4, TNF-α IL-1β and IFN-γ in Immune tolerant (IT), chronic hepatitis B (CHB), Inactive carriers (IC) and healthy controls (HC).

**Fig. S9**


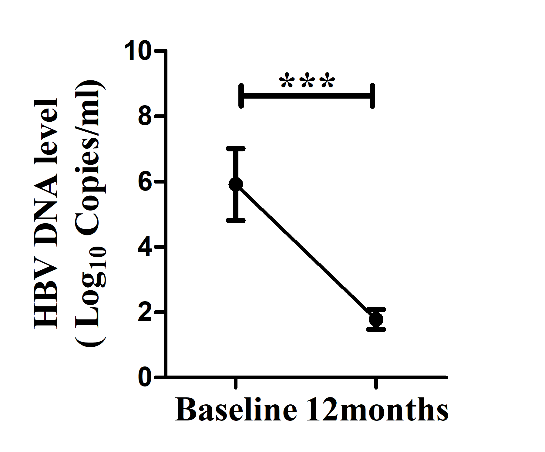

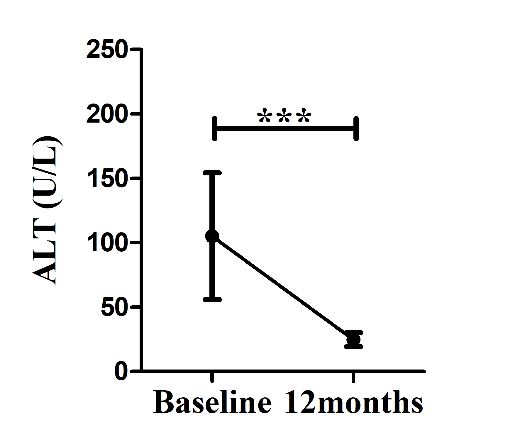


**Fig. S9.** Levels of HBV-DNA and ALT in sera of CHB patients at baseline and after 12 months of Tenofovir therapy. Paired t test was performed (****p* < 0.0001).

**Fig. S10**

**
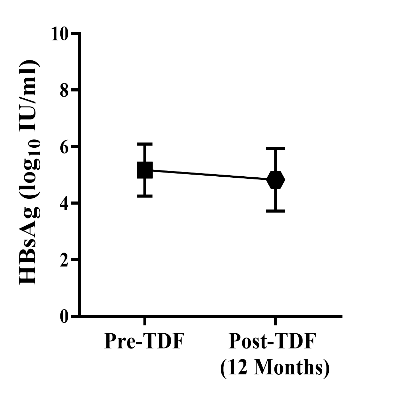

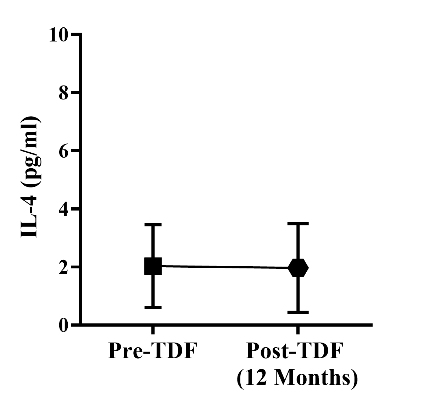

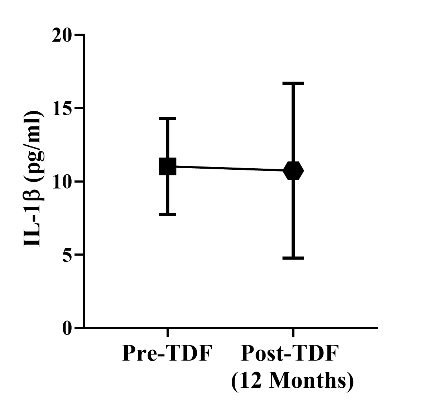
**

**Fig. S10.** Serum levels of HBsAg, IL-4 and IL-1β in CHB patients before initiation (pre-TDF) and after one year of antiviral Tenofovir therapy (post-TDF).

# **Key Reagent Table:**

| **Reagents** | | | **Source** | **Identifier** |
| --- | --- | --- | --- | --- |
| **Antibodies** | | | | |
| anti-human anti-C3-AF647 primary antibody | | | Santa Cruz Biotechnology | sc-28294 |
| mouse anti-human anti-C/EBPβ | | | Santa Cruz Biotechnology | SC7962 |
| mouse anti-human anti-C/EBPβ | | | Abclonal | A19538 |
| rabbit anti-human anti-p-CEBPβ | | | Cell Signaling Technology | 3084T |
| mouse anti-human anti- α tubulin | | | Santa Cruz Biotechnology | SC398103 |
| mouse anti-human anti-H3 primary antibody | | | Santa Cruz Biotechnology | SC517576 |
| anti-H3K9Ac antibody | | | Abclonal | A7255 |
| Horseradish peroxidase (HRP)-labelled goat anti-mouse IgG secondary antibody | | | Thermo scientific | 32430 |
| goat anti-rabbit IgG secondary antibody | | | Cell Signaling Technology | 7074P2 |
| mouse anti-HBsAg antibody | | | My Biosource | MBS430153 |
| goat anti-rabbit IgG-AF488 secondary antibody | | | Thermo Fisher Scientific | A11034 |
| donkey anti-mouse IgG-AF647 secondary antibody | | | Thermo Fisher Scientific | A31571 |
| mouse anti-HBX antibody | | | Invitrogen | MA5-47490 |
| mouse anti-HBsAg antibody | | | MyBiosource | MBS430153 |
| BD Pharmingen^TM^ FITC Mouse Anti-Human CD14 | | | BD Biosciences | 555397 |
| BD Horizon^TM^ BV421 Mouse Anti-Human HLA-DR | | | BD Biosciences | 562804 |
| BD Pharmingen^TM^ PECY^TM^7 Mouse Anti-Human CD3 | | | BD Biosciences | 557851 |
| BD^TM^ PerCP Mouse Anti-Human CD8 | | | BD Biosciences | 347314 |
| BD Pharmingen^TM^ FITC Mouse Anti-Human CD4 | | | BD Biosciences | 555346 |
| BD Horizon^TM^ BV421 Mouse Anti-Human IFN-γ | | | BD Biosciences | 562988 |
| BD PharmingenTM APC Mouse Anti-Human TNF | | | BD Biosciences | 551384 |
| PE anti-human IL-6 Antibody | | | Biolegend | 501106 |
| BD Pharmingen^TM^ PE Mouse Anti-Human IL12 (p40/p70) | | | BD Biosciences | 554575 |
| APC anti-human IL-10 Antibody | | | Biolegend | 506806 |
| BD Pharmingen^TM^ PE Rat Anti-Human IL2 | | | BD Biosciences | 560902 |
| **Chemicals, peptides, and recombinant proteins** | | | | |
| TRIzol Reagent | | THERMO | | 15596026 |
| RevertAid Reverse Transcriptase | | THERMO | | EP0442 |
| RNase inhibitor | | BIO BHARTI | | BB-X0010 |
| Random Hexamer Primer | | THERMO | | SO142 |
| PowerUp SYBR Master Mix | | Thermo | | A25742 |
| Lipofectamine 3000 Transfection Reagent | | INVITROGEN | | L3000-008 |
| Dulbecco’s Modified Eagle Medium (DMEM) | | HIMEDIA | | AL007A |
| Fetal Bovine Serum | | INVITROGEN | | 16000044 |
| L-Glutamine Solution | | SIGMA | | G7513-100ML |
| LguI(SapI) | | THERMO | | ER1932 |
| Dpn1 | | thermo | | ER1701 |
| Dimethyl sulfoxide for molecular biology (DMSO) | | SIGMA | | D8418-100ML |
| Poly-L-Lysine Solution | | SIGMA | | P8920-100ML |
| ProLong Gold Antifade Reagent with DAPI | | Cell Signaling Technology | | #8961 |
| 5-Aza-2-deoxycytidine | | SIGMA | | A3656 |
| Trichostatin A | | SIGMA | | T8552-1mg |
| cOmplete, Mini Protease Inhibitor Cocktail | | ROCHE | | 04693124001 |
| DL- Dithiothreitol (DTT) | | SIGMA | | D0632-1G |
| Phosphatase Inhibitor Cocktail | | SIGMA | | P0044 |
| Bradford Reagent | | SIGMA | | B9616-500ML |
| Immobilion-P PVDF Membrane | | MILLIPORE | | IPVH00010 |
| SuperSignal West Pico PLUS Chemiluminiscent Substrate | | INVITROGEN | | 34580 |
| G-418 Solution | | SIGMA | | 04727878001 |
| Lipopolysaccharides from *Escherichia coli* O111:B4 | | SIGMA | | L-4391 |
| Brefeldin A | | SIGMA | | B6542 |
| BD FACS^TM^ Lysing Solution 10X Concentrate | | BD Biosciences | | 349202 |
| BD Cytofix/Cytoperm^TM^ Fixation/Permeabilization kit | | BD Biosciences | | 554714 |
| HiSep™ LSM 1077 | | Himedia | | LS001 |
| RPMI 1640 Medium | | Invitrogen | | 11875093 |
| Pepset of 16 peptides, ACM Acid/Amide linker, 1-3mg (HBV core Overlapping Peptide Pool) | | Mimotopes | | PSACM0016-01 |
| HBsAg recombinant protein:: Hepatitis B Surface Antigen Adw subtype Recombinant Protein | | MyBioSource | | MBS142506 |
| Β-galactosidase | | SIGMA | | G5160 |
| Purified anti-IL-4 Monoclonal Antibody | | Elabsciences | | E-AB-F1203A |
| LEAF^TM^ Purified anti-human TNF-α | | Biolegend Inc | | 502803 |
| Anti-Hu IL-1 beta, eBioscience^TM^ | | invitrogen | | 14-7018-81 |
| Recombinant C3a protein | | PROSPEC | | PRO-2685 |
| **Commercial Kits** | | | | |
| Human C3(Complement Component 3) ELISA Kit | Elabscience | | | E-EL-H6054 |
| QIAquick Gel Extraction Kit | Qiagen | | | 28704 |
| Dual-Luciferase® Reporter Assay System | Promega | | | E1910 |
| EpiQuik™ Chromatin Immunoprecipitation Kit | EPIGENTEK | | | P-2002-1 |
| EZ methylation Gold Kit | ZYMO RESEARCH | | | D5005 |
| Favorgen DNA Extraction kit | Favorgen | | | FATGK001 |
| QIAamp DNA Blood Kits – Genomic DNA Extraction | Qiagen | | | 51104 |
| Human Th1/Th2 Cytokine Cytometric Bead Array (CBA) Kit II | BD Bioscience | | | 551809 |
